# Human papillomavirus E6/E7 oncoproteins promote radiotherapy-mediated tumor suppression by globally hijacking host DNA damage repair

**DOI:** 10.7150/thno.78091

**Published:** 2023-01-31

**Authors:** Diane Bruyere, Patrick Roncarati, Alizee Lebeau, Thomas Lerho, Florian Poulain, Elodie Hendrick, Charlotte Pilard, Celia Reynders, Marie Ancion, Margaux Luyckx, Michael Renard, Yves Jacob, Jean-Claude Twizere, Raphael Peiffer, Olivier Peulen, Philippe Delvenne, Pascale Hubert, Alison McBride, Nicolas Gillet, Murielle Masson, Michael Herfs

**Affiliations:** 1 Laboratory of Experimental Pathology, GIGA-Cancer, University of Liege, 4000 Liege, Belgium.; 2 Namur Research Institute for Life Sciences (NARILIS), Integrated Veterinary Research Unit (URVI), University of Namur, Namur, Belgium.; 3 Unit of Molecular Genetics of RNA Viruses, UMR 3569, CNRS, Pasteur Institute, University of Paris Diderot, 75015 Paris, France.; 4 Laboratory of Signaling and Protein Interactions, GIGA-Molecular Biology of Diseases, University of Liege, 4000 Liege, Belgium.; 5 Metastasis Research Laboratory, GIGA-Cancer, University of Liege, 4000 Liege, Belgium.; 6 Department of Pathology, University Hospital of Liege, 4000 Liege, Belgium.; 7 Laboratory of Viral Diseases, National Institute of Allergy and Infectious Diseases, National Institutes of Health, Bethesda, MD, 20892, USA.; 8 Biothechnology Superior School, UMR 7242, CNRS, University of Strasbourg, 67412 Illkirch, France.

**Keywords:** human papillomavirus, DNA damage and repair, radiotherapy, protein-protein interactome.

## Abstract

**Rationale:** Whatever the mucosa primary infected, HPV-positive cancers are traditionally associated with a favorable outcome, attributable to a high sensitivity to radiation therapy. However, the direct impact of viral E6/E7 oncoproteins on the intrinsic cellular radiosensitivity (and, globally, on host DNA repair) remains mostly speculative.

**Methods:** Using several isogenic cell models expressing HPV16 E6 and/or E7, the effect of viral oncoproteins on global DNA damage response was first investigated by *in vitro/in vivo* approaches. The binary interactome of each individual HPV oncoprotein with factors involved in the various host DNA damage/repair mechanisms was then precisely mapped by *Gaussia princeps* luciferase complementation assay (and validated by co-immunoprecipitation). The stability/half-life of protein targets for HPV E6 and/or E7 as well as their subcellular localizations were determined. At last, the host genome integrity following E6/E7 expression and the synergy between radiotherapy and compounds targeting DNA repair were analyzed.

**Results:** We first showed that the sole expression of one viral oncoprotein from HPV16 was able to significantly increase the sensitivity to irradiation of cells without affecting their basal viability parameters. In total, 10 novel targets (CHEK2, CLK2, CLK2/3, ERCC3, MNAT1, PER1, RMI1, RPA1, UVSSA and XRCC6) for E6 and 11 (ALKBH2, CHEK2, DNA2, DUT, ENDOV, ERCC3, PARP3, PMS1, PNKP, POLDIP2 and RBBP8) for E7 were identified. Importantly, not degraded following their interaction with E6 or E7, these proteins have been shown to be less linked to host DNA and to colocalize with HPV replication foci, denoting their crucial implication in viral life cycle. Finally, we found that E6/E7 oncoproteins globally jeopardize host genome integrity, increase the cellular sensitivity to DNA repair inhibitors and enhance their synergy with radiotherapy.

**Conclusion:** Taken together, our findings provide a molecular insight into the direct hijacking of host DNA damage/repair responses by HPV oncoproteins, demonstrate the significant impact of this phenomenon on both intrinsic cellular radiosensitivity and host DNA integrity and suggest novel connected therapeutic vulnerabilities.

## Introduction

Although over 250 million doses of human papillomavirus (HPV) vaccine have already been distributed since its introduction 15 years ago, carcinogenic HPV genotypes (most notably HPV16) are still responsible for an estimated 690,000 cancers per year worldwide 1. Diagnosed in the anogenital and upper aero-digestive tracts, HPV-driven tumors are indisputably associated with a better outcome than their HPV-uninfected counterparts 2-6, which led to both the recent down-staging of patients with oropharyngeal HPV-positive squamous cell carcinoma (SCC) (AJCC, 8th edition) and the clinical evaluation of several de-intensification treatment strategies (e.g., reduced-dose radiotherapy) (for a review, see 7). Whatever the primary site of infection, and despite the growing interest for targeted therapies or immune checkpoint inhibitors, the recommended first-line treatment modalities for HPV-related neoplasms still (in the large majority of cases) involves radiation therapy (fractionated exposure to 45-70 Gy), chemotherapy (most often with platinum-derived compounds) and surgical excision (when possible).

The main factors determining the success or failure of radiotherapy were first explained in the mid 70's by Prof. Rodney Withers in a seminal article entitled “The four R's of radiotherapy”. The repopulation of surviving normal and tumor cells between dose fractions, redistribution of malignant cells in the G2/M cell cycle phase, reoxygenation of the hypoxic areas and repair of DNA damage induced by ionizing radiation were originally proposed. A few months later, the intrinsic radiosensitivity of irradiated cells was presented as the fifth “R”. At last, a “6R” model has been recently suggested, adding the reactivation of immune responses to the five aforementioned R's 8. In the context of HPV-positive cancers, three main parameters are commonly proposed to explain the favorable prognosis/increased sensitivity to radiation therapy: the wild-type status of *TP53* 9, the elevated T-cell density detected within tumor microenvironment 10-12 and the low tumor hypoxia 13. In parallel, data accumulated in the last few years indicate that HPV could also directly participate to this enhanced radiation sensitivity by disrupting DNA damage/repair responses (particularly the canonical non-homologous end-joining, homologous recombination and Fanconi anemia DNA repair pathways) 14-17. Still frequently disregarded by both clinicians and researchers, the primary goal of the virus is to complete its life cycle and not to promote carcinogenesis (which ultimately impedes its amplification and kills its host). To do so, and beside the requirement of sustaining keratinocyte proliferation, it becomes obvious that HPV must hijack the host signaling cascades to promote rapid repair and faithful replication of its genome. As a side effect, host DNA would become more vulnerable to the acquisition of genomic alterations, leading to (pre)cancer development. The molecular binding partners of viral oncoproteins involved in this process are, however, still mostly unknown. Of note, indirect mechanisms involving p16^ink4a^ (a surrogate biomarker for HPV infection) or the defect of TGFβ signaling were also reported to explain the enhanced radiosensitivity of HPV-positive tumors. While the down-regulation of E3 ubiquitin ligase TRIP12 has been demonstrated to be essential in p16^ink4a^-mediated repression of DNA damage/repair 18, 19, conflicting results exist regarding the HPV-TGFβ interplay and the subsequent miR-182-dependent inhibition of BRCA1, required for homologous recombination repair 20, 21.

Altogether, this context opens quite a few theoretical and translational questions. Could HPV-related “manipulation” of host DNA repair mechanisms represent the Achilles' heel of HPV-positive cancers? Are viral oncoproteins alone able to sensitize infected cells to irradiation-induced DNA single/double-strand breaks? Do E6 and E7 directly interact with key proteins involved in DNA damage repair pathways? Is the function, half-life and/or cellular localization of DNA repair proteins altered by viral oncoproteins? Could E6 and E7 oncoproteins enhance the sensitivity to DNA repair inhibitors and/or positively impact their synergy with radiotherapy? By using various *in vitro*/*in vivo* models as well as by mapping the interactome of HPV E6 and E7 oncoproteins with the different families of DNA repair proteins, the present study attempts to answer all these questions.

## Materials and Methods

### Human tissue specimens

A total of 59 oropharyngeal and 113 anal canal SCC were retrieved from pathology archives of the University Hospital of Liege (Belgium). Before selection, all cases were re-examined by experienced histopathologists. The paraffin-embedded specimens were processed and archived in the local Biobank throughout the project. For each specimen, the HPV status was determined by both immunohistochemistry (anti-p16^ink4a^) and HPV DNA genotyping [Abbott RealTime High-Risk HPV assay (Abbott, Wiesbaden, Germany)]. The study was approved by the institutional review board at the University Hospital of Liege (#2021/25).

### Cell lines and culture conditions

Eleven HPV-negative and 7 HPV-positive cell lines were maintained in Dulbecco's modified Eagle's medium (DMEM), Minimum Essential medium (MEM) or Roswell Park Memorial Institute (RPMI) 1640 medium (Gibco, Thermo Fisher Scientific, Waltham, MA, USA) supplemented with 10% fetal calf serum and various additives. Precise cell culture conditions are detailed in Table S1. With the exception of U-2OS (osteosarcoma), HEK-293T (embryonic kidney) and HaCaT cells (immortalized keratinocytes), all cell lines were derived from the gynecologic or upper aerodigestive tracts. SiHa (HPV16), CaSki (HPV16), C4-II (HPV18), MDA-1483 (HPV18), HT-3 (HPV30), HeLa (HPV18), UPCI-SCC-154 (HPV16) as well as UPCI-SCC-40, UPCI-SCC-111, FaDu, SQ-20B, UD-SCC-1, CAL-27, CAL-33, BHY, A431, UPCI-SCC-036, UPCI-SCC-114 (HPV-negative) were obtained from ATCC or DSMZ (German collection of microorganisms and cell cultures). All cell lines used in this study have been regularly tested for potential mycoplasma contamination [MycoAlert Mycoplasma Detection kit (Lonza, Basel, Switzerland)] and were invariably found to be negative.

### Isogenic cell models expressing HPV16 E6 and/or E7

HPV-negative malignant vulvar (A431), floor of mouth (UPCI-SCC-111) and osteosarcoma (U-2OS) cells (previously used as a model for studying HPV-related carcinogenesis 22, 23) as well as immortalized/noncancerous keratinocytes (HaCaT) were stably transduced with HPV16 E6 and/or E7 or firefly luciferase (negative control) (GIGA-Viral Vectors, University of Liege). Using pSPAX2 (Addgene, Watertown, MA, USA), pLV-EF1a-IRES Luciferase plasmid (VectorBuilder, Chicago, USA), either empty or containing the coding sequence for HPV16 E6 or E7, was co-transfected with a VSV-G-encoding vector into Lenti-X 293T cells (Clontech, Mountain View, USA). Forty-eight and 72 h post-transfection, viral supernatants were collected, concentrated, purified and titrated (qPCR Lentivirus Titration kit; ABM, Richmond, Canada). Transduced cells were finally selected with 100 µg/ml hygromycin B or 10 µg/ml blasticidin (InvivoGen, San Diego, CA, USA).

### In vivo mouse irradiation model

A431 Luc or A431 E6E7 cells (7.5x10^5^ in 150-300 µl of DMEM) were subcutaneously injected (left flank) in Nude mice aged 4-6 weeks (n = 13 per condition). Throughout the experiment, tumor size was monitored every 2-3 days with a digital caliper (Thermo Fisher Scientific). When tumor volume [π /6 x (length × width^2^)] reached 100-200 mm^3^, a unique dose of 6 Gy was administrated using a small animal irradiator (X-RAD 225Cx; Precision X-Ray, North Branford, CT, USA). At day 9 after irradiation, the treated animals were anesthetized and 20 mg/kg luciferin (Promega) was injected intraperitoneally. Ten minutes later, mice were imaged using an IVIS^®^ Lumina III *in vivo* Imaging System (PerkinElmer, Waltham, MA, USA). For ethical reasons, mice were euthanized when the average tumor volume exceeded 1000 mm^3^ in the control group. All animals were purchased from Janvier Labs (Le Genest-Saint-Isle, France) and the authors strictly complied to the ethical recommendations established by the Federation of European Laboratory Animal Sciences Associations (FELASA). The procedures were initially reviewed and approved by the local ethics committee (#19-2109).

### Immunohistochemistry and immunostaining assessment

Immunohistochemical experiments were performed as previously described 2, 24-26. Briefly, slides were first deparaffinized in xylene, rehydrated in graded alcohol and endogenous peroxidases were inhibited using 4.5% H_2_O_2_ in methanol for 5 min. Antigens were retrieved in 10 mM citrate buffer (pH6) for 23 min at 100 °C. Before the primary reaction (1 h at room temperature), non-specific antigens were blocked using serum-free protein block reagent (Dako, Glostrup, Denmark) for 10 min. The following primary antibodies were used: anti-p16^ink4a^ (1/100, ENZ-ABS377-0100, Enzo Life Sciences, Farmingdale, NY, USA) and anti-γH2AX (1/100, clone 20E3, Cell Signaling Technology, Danvers, MA, USA). For the immunoperoxidase staining, the rabbit EnVision detection kit (Dako) was used and positive cells were visualized using SignalStain DAB Substrate kit (Cell Signaling Technology).

All immunolabelled tissues were evaluated independently by two histopathologists. As previously described 2, p16^ink4a^ staining was considered as positive when >75% cancer cells strongly expressed this surrogate biomarker for HPV infection. The number of γH2AX-positive tumor cells per mm^2^ was precisely determined using QuPath 0.2.0 software for digital pathology image analysis (computerized counting) 27.

### TP53 mutation analysis

Genomic DNA was extracted from cultured cells using the NucleoSpin Tissue kit (Macherey-Nagel, Düren, Germany). Exons 2 to 11 of *TP53* were amplified by classical PCR. The primer sequences were previously described 24. The PCR products were then purified and sequenced using an ABI 3700 automated sequencer (Applied Biosystems, Foster City, CA, USA) (GIGA-Genomics platform, University of Liege).

### Gene expression analysis (using public dataset)

The expression levels of *ALKBH2, BRCA1, BRCA2, CHEK2, CLK2, DNA2, DUT, ENDOV, ERCC3, H2AX, MNAT1, PARP3, PER1, PMS1, PNKP, POLDIP2, RAD51, RBBP8, RMI1, RPA1, UVSSA, XRCC1, XRCC2, XRCC3, XRCC4, XRCC5* and *XRCC6* in head and neck cancers were evaluated using The Cancer Genome Atlas (TCGA) public dataset through the cBioPortal interface 28. This published dataset was also used to run a gene set enrichment analysis (GSEA) using both the KEGG pathways and GO database. GSEA was performed using RStudio v1.4.1 (clusterProfiler 4.0 package) 29, 30. In parallel, data related to the overall survival were retrieved. All samples were separated according to their HPV status.

### Quantitative reverse transcription PCR (RT-qPCR)

Total RNA was extracted and purified from cultured cells using the NucleoSpin RNA isolation kit (Macherey-Nagel). One µg was then reverse transcribed using RevertAid Reverse Transcriptase and oligodT primers (Thermo Fischer Scientific). qPCR experiments (QuantStudio 3, Applied Biosystems) were performed using the FastStart Universal SYBR Green Master mix (Roche, Basel, Switzerland) and the following primers sequences: HPV16 E6 forward: 5'-TGG AAT CTT TGC TTT TTG TCC-3'; E6 reverse: 5'-CTG CGA CGT GAG GTG TAT TAA C-3'; HPV16 E7 forward: 5'-GGT TAC AAT ATT GTA ATG GGC TC-3'; E7 reverse: 5'-AGC TCA GAG GAGGAG GAT GAA-3'; GAPDH forward: 5'-ACC AGG TGG TCT CCT CTG AC-3'; GAPDH reverse: 5'-TGC TGT AGC CAA ATT GGT TG-3'. Each experiment was performed in triplicate and normalized to the amount of GAPDH mRNA from the same sample.

### Cell Proliferation

Cells were seeded in order to reach ~10% confluence. After cell adhesion, 24-well plates were incubated for 7 days in the IncuCyte S3 Live-Cell Analysis System (Sartorius, Göttingen, Germany). Every 12 h, pictures were taken (4 fields per well). Collected data were finally analyzed using the IncuCyte S3 software.

### Apoptosis

The percentage of apoptotic cells was determined by flow cytometry (FACSCalibur flow cytometer, BD Biosciences, Franklin Lakes, NJ, USA), using annexin V-FITC and propidium iodide, according to the manufacturer's recommendations (BD Biosciences).

### Cell cycle analysis

Cells were harvested using trypsin-EDTA solution, washed with PBS and fixed with iced cold 70% ethanol overnight at -20 °C. Fixed cells were then washed once with PBS and incubated for 30 min at room temperature with a solution containing RNase A (50 µg/ml, Macherey-Nagel, Germany) and propidium iodide (50 µg/ml, Invitrogen, Carlsbad, CA, USA). Cells were finally analyzed by flow cytometry (FACSCalibur flow cytometer, BD Biosciences).

### Neutral comet assay

Cells were first irradiated with 40 Gy (Gammacell 40 Exactor, Best Theratronics, Vancouver, Canada) at room temperature and incubated for the indicated times (0 h, 1 h, 3 h and 6 h) at 37 °C in order to allow for DNA repair. Cells were then collected and processed for neutral comet assay as extensively described previously 31. Briefly, OxiSelect comet slides (3-well slides, Cell Biolabs, San Diego, CA, USA) were used and cell lysis was performed overnight at 37 °C in a buffer containing 2% sarkosyl, 0.5M Na_2_EDTA and 0.5 mg/ml proteinase K (pH 8.0). Electrophoresis was conducted for 25 min at 0.6 V/cm in a solution containing 90 mM Tris, 90 mM boric acid and 2 mM Na_2_EDTA (pH 8.5). Following an incubation with Vista Green DNA dye for 20 min, comets were detected, photographed (6 fields per well) using a fluorescence microscope (Vanox AH BT3, Olympus, Tokyo, Japan) and quantified (CometScore 2.0 software). Tail length was used to estimate the degree of DNA damage (double-strand breaks) in each condition.

### Cytokinesis-block micronucleus assay

Plated on a glass coverslip, 5 x 10^4^ cancer cells transduced with HPV16 E6 and/or E7 or firefly luciferase (control) were exposed to 0, 2 or 4 Gy irradiation. After a 4 h recovery, cytochalasin B (3 μg/ml, Sigma Aldrich), a potent cytokinesis inhibitor, was added to the media for 18 h. The cells were then quickly washed with PBS, fixed with 4% paraformaldehyde (15 min at room temperature) and stained with 4′,6-diamidino-2-phenylindole (DAPI). Micronucleus frequency was finally determined in once-divided binucleated cells according to previously described criteria 32.

### Clonogenic growth analysis

HPV-negative and -positive cells were seeded in 6-well plates at a density comprised between 250 and 2,000 cells per well, depending on their growth rate. After 24h, cells were irradiated with 0, 1, 2, 4 or 6 Gy using a Gammacell 40 Exactor (Best Theratronics) and incubated for 10 days at 37 °C in a humidified 5% CO_2_ atmosphere. Plates were then washed in PBS, fixed and colored for 45 min using a solution containing 5% glutaraldehyde (Acros Organics, Thermo Fischer scientific) and 0,5% Crystal violet (Sigma-Aldrich, Saint Louis, MI, USA). After a washing step with deionized water, plates were dried, scanned (Epson Perfection V500 PHOTO, Epson, Nagano, Japan) and quantified using the ColonyArea ImageJ plugin (ImageJ software, National Institute of Health, Bethesda, MD, USA). Instead of the traditional manual counting, this standardized/computerized approach allows to determine the percentage of area covered by cell colonies 33.

### Plasmid library generation

Both derived from pCiNeo vector, pSpica-N1 and pSpica-N2 plasmid vectors express Gluc1 and Gluc2, respectively. These complementary fragments of the *Gaussia princeps* luciferase are linked to the N-terminal ends of the proteins of interest through a 20-amino acid flexible hinge region. pSpica-N2 vectors expressing E6 or E7 from different high-risk (carcinogenic) HPV genotypes (HPV16, 18, 33 and 39) were previously generated 34. Open reading frames (ORFs) encoding for proteins involved in DNA repair mechanisms were obtained from the human ORFeome collections v7.1 and 8.1 (Dana-Farber Cancer Institute, Boston, USA). Initially contained into a pDONR223 entry vector, the ORFs were transferred into the pSpica-N1 destination vector *via* Gateway cloning. In order to verify both the cloning step and the accuracy of all transferred sequences, resulting plasmids were sequenced (Sanger sequencing, GIGA-Genomics platform, University of Liege) using the following forward primer: 5'-CAG CTC TTA AGG CTA GAG TAC-3'. A library of 200 pSpica-N1 plasmids coding for 179 DNA damage and repair proteins (and 21 isoforms) was generated (Table S2).

### Gaussia Princeps luciferase Complementation Assay (GPCA)

Twenty-four hours before transfection, 3x10^4^ HEK-293T cells were seeded per well of a Costar flat bottom white 96-well plate (Corning Life Sciences, Amsterdam, The Netherlands). Cells were transfected with 100 ng pSpica-N2-HPV E6 or E7 and 100 ng pSpica-N1 (from the DNA repair library) using PEImax (Polysciences, Warrington, PA, USA). Twenty-four hours post-transfection, cells were washed and then incubated for 30 min at room temperature with the Renilla lysis buffer (E2820, Promega, Madison, WI, USA). After injection of 50 µl of luciferase substrate reagent in each well, the luciferase activity was measured during 10 s using a Centro LB960 microplate luminometer (Berthold Technologies, Bad Wildbad, Germany). As previously described 34, 35, results were expressed as relative luminescence units (RLU) or as a normalized luminescence ratio (NLR). NLR was calculated by dividing the RLU by the sum of controls. NLR= (Gluc1-A and Gluc2-B) / [(Gluc1-empty and Gluc2-B) + (Gluc1-A and Gluc2-empty)].

### Co-immunoprecipitation (co-IP)

HPV16 E6 or E7 sequence was first transferred into the pCineo-3xFLAG vector, allowing the expression of a 3xFLAG tag at the N-terminal end of the viral oncoproteins. The day before transfection, 4x10^5^ HEK-293T cells per well of a 6-well plate were seeded. Cells were transfected with 1.5 µg pCineo-3xFLAG (HPV16 E6, HPV16 E7 or empty) and 1.5 µg pSpica-N1 from our library using PEImax (Polysciences). Twenty-four hours post-transfection, cells were harvested and incubated for 30 min on ice in 100 µl lysis buffer (50 mM Tris-HCl pH7.4, 150 mM NaCl, 1 mM EDTA, 1%Triton X-100 and proteases inhibitors). After a centrifugation step (18,900 g for 30 min at 4 °C), 96 µl collected supernatant was incubated overnight with 30 µl anti-Flag M2 Magnetic Beads (Sigma-Aldrich). The 4 remaining µl represent the total fraction of the extraction. Total and immunoprecipitated proteins were analyzed by western blotting. The nitrocellulose membranes were incubated overnight at 4 °C with anti-*Gaussia luciferase* antibody (E8023, New England Biolabs, Ipswich, MA, USA). After several washings, the membranes were incubated for 1 h at room temperature with a secondary goat anti-rabbit antibody (G21234, Invitrogen). The protein bands were finally revealed using a chemiluminescence system (Pierce ECL substrate, Thermo Fisher Scientific).

### Subcellular protein fractionation and western blotting

The harvested cells were first incubated in cytoplasmic buffer (HEPES pH 7.4 10 mM, KCl 10 mM, MgCl_2_ 2 mM, EDTA 0.1 mM, NP-40 0.2%, DTT 1 mM and protease inhibitors) for 30 s. After centrifugation (4,000 g for 5 min at 4 °C), the cytoplasmic fraction (supernatant) was collected. The pellet was then washed 5 times (washing buffer: HEPES pH 7.4 10 mM, KCl 20 mM, MgCl_2_ 2 mM, EDTA 0.1 mM, DTT 1 mM and protease inhibitors) before being lysed in buffer B (EDTA 3 mM, EGTA 0.2 mM, DTT 1 mM and protease inhibitors) for 30 min. The fraction corresponding to the nucleic acid-free proteins was collected after centrifugation (320 g for 7 min at 4 °C). Finally, the proteins linked to the chromatin were isolated after sonication (30 s) in Laemmli buffer (Tris-HCl pH8 62.5 mM, SDS 2%, glycerol 10% and protease inhibitors) at 4 °C. After quantification (BCA protein assay; Pierce, Rockford, IL, USA), 30 µg of proteins were separated by electrophoresis (SDS-PAGE) and transferred onto PVDF membranes (Roche), that were blocked with 5% nonfat milk in TBS-tween 0.1%, before being incubated overnight at 4 °C with the primary antibodies (listed in Table S3). The membranes were then incubated with an anti-rabbit (G21234, Invitrogen) or anti-mouse (P0260, Dako) secondary antibody for 1 h. The protein bands were detected using an enhanced chemiluminescence system (Pierce ECL substrate, Thermo Fisher Scientific) and finally quantified by densitometric analysis (ImageJ software). To validate the fractionation method, anti-MEK2 (9125S; Cell Signaling), anti-YY1 (sc-7341; Santa Cruz Biotechnology, Dallas, TX, USA) and anti-HDAC2 (sc-9959; Santa Cruz Biotechnology) antibodies were used (positive controls).

### Cycloheximide chase assay

HaCaT cells transduced with HPV16 E6, E7 or firefly luciferase (negative control) were treated with 100 µM Cycloheximide (Sigma Aldrich). The proteins were isolated in SDS buffer (SDS 1%, Tris-HCl pH 7.5 40 mM, EDTA 1 mM, protease inhibitors) at different time points (up to 24 h) and then quantified (BCA protein assay; Pierce). The level of given proteins was finally determined by western blot (see Table S3 for primary antibody specificities). Anti-actin (A5441; Sigma-Aldrich) and anti-HSC-70 (sc-7298; Santa Cruz Biotechnology) antibodies were used for normalization.

### Recircularization of HPV16 genome

Full-length HPV16 DNA was first released from the pUC19 plasmid using FastDigest BamHI restriction enzyme, according to the manufacturer's instructions (Thermo Fisher Scientific). The linear genome was then recircularized by incubating 10 µg of the digested plasmid with 400 U of T4 ligase, 10X ligation reaction buffer and RNase free water (New England Biolabs, Ipswich, MA, USA) overnight at 16 °C. Finally, DNA was precipitated (NaCl 5 M and isopropanol at -20 °C), centrifuged (16 000 g for 30 min at 4 °C) and resuspended in TE buffer (Tris 10 mM, EDTA 1 mM).

### HPV16 E1 and E2 transductions and immunofluorescence

HPV-negative malignant vulvar (A431) cells were stably transduced with HA-tagged HPV16 E1 and 3xFlag-tagged HPV16 E2 or mCherry (negative control) (GIGA-Viral Vectors, University of Liege), as described above (see Isogenic cell models expressing HPV16 E6 and/or E7). Given that the constitutive expression of E1 and E2 proteins is lethal for the cells, we used the Double-Floxed Inverted Open reading frame (DIO) technology to invert the Open Reading Frame (ORF). Therefore, the E1 and E2 genes are not expressed unless a Cre treatment is applied to the cell culture. Transduced cells were selected with 133 µg/ml hygromycin B or 0.33 µg/ml puromycin (InvivoGen).

Coverslips were coated with 0.1% poly-l-lysine (15 min at room temperature), sterilized using UV light and distributed in 6-well plates. Then, 1.25x10^5^ E1/E2-transduced cells were seeded per well. After 24 h, cells were transiently transfected with 0.5 µg of the recircularized HPV16 genome using FuGene 6 (Roche), according to the manufacturer's instructions. Six hours later, a non-integrative Cre lentivirus was added to the cell culture in order to induce the expression of HPV16 E1 and E2, by flipping the ORF to the right direction. Thirty-six hours after the Cre induction, the cells were quickly washed with PBS, fixed with 4% paraformaldehyde (15 min at room temperature), permeabilized with PBS-0.1% triton X-100 (15 min) and blocked with an animal-free blocking solution (cell signaling, 20 min). The cells were then incubated with the primary antibodies overnight at 4 °C [for additional information (e.g., clone numbers, dilutions), see Table S3]. After a washing step (PBS), the cells were incubated with conjugated (AlexaFluor 488 or AlexaFluor 647) anti-mouse and anti-rabbit secondary antibodies (1/1000) for 1 h at room temperature. Where indicated, 1h before the fixation step, the cells were treated with 10 µM 5-ethynyl-2'-deoxyuridine (EdU) (Click-iT™ Plus EdU Cell Proliferation Kit for Imaging, Thermo Fisher Scientific), a thymidine analog which is incorporated into DNA and used to evaluate DNA synthesis. EdU staining was performed according manufacturer's instructions. Finally, the coverslips were washed (PBS), mounted with Fluoromount-G™ Mounting Medium (Thermo Fischer Scientific) and visualized using a Nikon Eclispe Ti microscope (Magnification: X1000). Colocalization analysis was performed using the JACoP ImageJ plugin (ImageJ software).

### Whole genome sequencing

HPV16 E6E7 isogenic cell pairs generated from immortalized keratinocytes (HaCaT) were grown (at 37 °C in a humidified 5% CO_2_ atmosphere) until a ~80% confluence was reached. The cell culture medium and supplements are detailed in Table S1. At each passage, the cells were detached by adding 0.25% trypsin-EDTA (Gibco), counted and 1 x 10^6^ cells were seeded into T75 flasks. At passage (P) 0, 10 and 20, expanded cells were harvested and genomic DNA was extracted using NucleoSpin Tissue kit (Macherey Nagel, Düren, Germany). DNA quantification [Quant-iT PicoGreen dsDNA Kit (Invitrogen)], library preparation (Illumina DNA PCR-Free Library Prep Kit using 25 ng DNA) and quantification/normalization [KAPA Library Quantification Kit (KapaBiosystems, Wilmington, MA, USA)] were then performed at the GIGA-Genomics platform (University of Liege). Sequencing (30X coverage) was conducted on the Illumina NovaSeq6000 platform (PE150 mode). Sequencing reads were aligned to the reference human genome (GRCh38) using the Burrows-Wheeler Alignment tool, with “bwa mem” settings 36. Bam files were formulated using the set of utilities SAMtools.

### Variant calling and mutational signature analysis

Genomic variants have been assessed/detected using the open-source Strelka2 software, with default settings 37. Both P10 and P20 samples were compared to the corresponding P0 read alignment sample. Only single nucleotide polymorphisms (SNPs) and insertions/deletions (InDels) filtered by Strelka2's empirical variant scoring (EVS) model were selected for both absolute/relative genomic alteration quantifications and mutational signatures analysis. The assignment of distinct mutational signatures reported in the Catalogue of Somatic Mutations in Cancer (COSMIC v3.3, June 2022) was performed using the SigProfilerExtractor package 38.

### Drug response curves

Potent/selective inhibitors of PARP1/2 (Niraparib and Veliparib), RAD51 (RI-1), homology-dependent DNA repair (YU238259) and DNA topoisomerase I (Camptothecin) were purchased from Selleckchem (Houston, TX, USA). For IC_50_ determination, eleven dilutions of each compound were tested. The cells were seeded at 3,000 (A431), 5,000 (HaCaT) or 8,000 (UPCI-SCC-111) cells per well in 96-well plates containing working dilution of each DNA repair inhibitor and incubated for 72 h at 37 °C. Cell viability was assessed by MTT proliferation assay (Roche). The individual IC_50_ values of each drug against the HPV E6E7 isogenic cell pair were calculated using nonlinear regression analysis (GraphPad Prism 8 software, San Diego, CA, USA).

### Combination treatments and synergy quantification

Both control and E6E7-trasnduced HaCaT cells (5,000 cells per well in 96-well plates) were exposed to growing doses of irradiation (0, 2, 4, 6, 8, 10 Gy) and/or to different concentrations (lower than IC50) of Niraparib, Veliparib, RI-1, Camptothecin or YU238259. After 72 h, cell viability was assessed by MTT proliferation assay (Roche). Synergy between radiotherapy and each DNA repair inhibitor was evaluated according to the Zero Interaction Potency (ZIP) method and using the open-source SynergyFinder 3.0 software 39, 40.

### Statistical analysis

Statistical significance between 2 or more groups/conditions was assessed using GraphPad Prism 8 software (San Diego, CA, USA). Normal distribution was determined by both the skewness score and a D'Agostino and Pearson normality test. The outlier identification was performed using the ROUT method, with a Q=5% 41. When two groups needed comparison, an unpaired t-test was performed whereas when more than two groups or conditions were compared, a one-way ANOVA was applied (followed by a Bonferroni post-test or by Dunnett's multiple comparison test). The comparison of cell cycle profiles (G1, S, G2/M) between independent groups was performed using a χ^2^ test. *p < 0.05, **p < 0.01, ***p < 0.001 and ****p < 0.0001.

## Results

### HPV-positive cancers highly express DNA damage response factors and display an elevated sensitivity to radiation therapy

**Figure 1:**
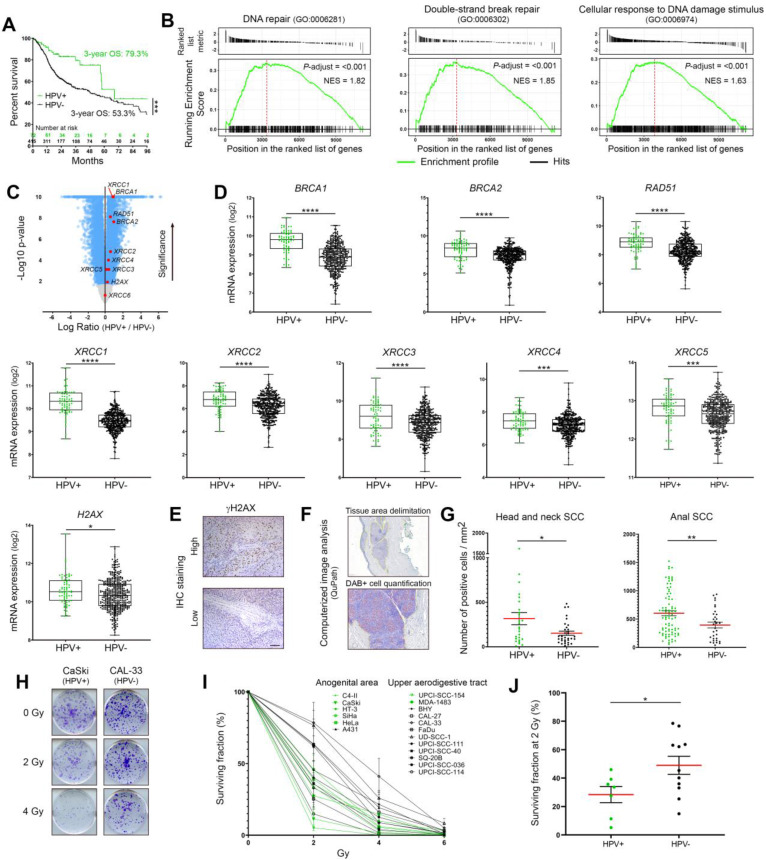
** Expression profiles of DNA damage response factors and radiosensitivity of HPV-negative and -positive cancers. (A)** Kaplan-Meier estimates of overall survival (OS) according to the HPV status (positive: n = 72; negative: n = 415). The TCGA dataset for head and neck neoplasms was used. **(B)** GSEA analyses of differentially expressed genes in HPV-positive *versus* HPV-negative tumors. The published dataset for head and neck tumors (TCGA) was used. NES: normalized enrichment score with adjusted *p*-value for each enrichment plots. **(C)** Volcano plot (x-axis: log_2_ HPV+/HPV- ratio; y-axis: -log_10_ *p*-value) showing the genes differentially expressed in HPV-positive SCC compared to their uninfected counterparts (blue dots, cut-off: *p* < 0.01). Standard DNA damage response biomarkers are highlighted in red. **(D)** *BRCA1, BRCA2, RAD51, XRCC1* to *5* and *H2AX* expression levels in head and neck cancers according to the HPV status. **(E)** Representative pictures of SCC displaying a high or low γH2AX expression. **(F)** Illustration of the computerized DAB-positive cell quantification (QuPath). **(G)** γH2AX-expressing cells were detected in each cancer tissue specimen (oropharyngeal or anal SCC) and the number of positive cells was reported to tumor area (mm^2^). The mean (in red) ± SEM is shown. **(H)** Representative pictures (at day 10 post-irradiation) of crystal violet-stained CaSki (HPV16) and CAL-33 (HPV-negative) cell colonies irradiated with 0, 2 or 4 Gy. **(I)** Percentage of area covered by cell colonies from HPV-negative (black) and HPV-positive (green) cell lines following treatment with growing doses of irradiation (0-6 Gy). For each cell line, the non-irradiated condition (0 Gy) was used as control and set to 100%. Results represent the means ± SEM of at least three independent experiments. **(J)** Clonogenic growth analysis data at 2 Gy for each analyzed cell line. The means of at least three independent experiments are shown. Results were separated into two groups based on the HPV status of cancer cells. The scale bar represents 100 μm. Asterisks indicate statistically significant differences (*p < 0.05, **p < 0.01, ***p < 0.001, ****p < 0.0001). *P* values were determined using an unpaired t-test (D, G, J).

By using the TCGA public dataset for head and neck SCC, both the overall survival and the mRNA levels of the most frequently used DNA damage/repair biomarkers (BRCA1, BRCA2, RAD51, XRCC1 to 6 and H2AX) were first assessed. A pathway enrichment analysis (GSEA) was also performed. All collected data were separated according to the HPV status (positive: n = 72; negative: n = 415). As expected, [and reported in previous publications 4, 5, patients with HPV-positive neoplasms were associated with a favorable outcome (Figure 1A). Interestingly, GSEA analysis revealed the significant enrichments of various gene signatures directly related to DNA damage/repair mechanisms in HPV-positive cancers compared to their HPV-negative counterparts (Figure 1B and Table S4). Many other gene sets, secondarily linked to DNA repair/maintenance (e.g., DNA helicase activities, cell cycle checkpoints…) have also been found to be enriched in viral-related neoplasms (Table S4). Supporting these results, with the exception of XRCC6, all genes coding for “classical” biomarkers of DNA repair activation were significantly more expressed in HPV-related cancers (Figure 1C-D). The protein level of phosphorylated H2AX on its serine 139 [active form, also called γH2AX (sensitive marker of DNA double-strand breaks)] was also analyzed by immunohistochemistry and a similar significant up-regulation was detected in both anal and oropharyngeal HPV-driven tumors (Figure 1E-G). Clonogenic growth analyses were then performed to determine the radiosensitivity of many HPV-negative (n = 11) and -positive (n = 7) cell lines from various origins (uterine cervix, vulva, head and neck). Ten days after being exposed to growing doses of irradiation (ranging from 0 to 6 Gy), the percentage of area covered by cell colonies was precisely determined by computerized counting. As shown in Figure 1H-J, despite some degree of heterogeneity within the two groups, overall, HPV-positive cells were significantly more radiosensitive than their HPV-unrelated counterparts. Containing about 600 copies of HPV16 per cell, it is interesting to notice that CaSki has been shown to be especially sensitive to radiation therapy (Figure 1H-I).

### E6/E7 viral oncoproteins increase the intrinsic radiosensitivity of cells independently of affecting their basal viability parameters

**Figure 2:**
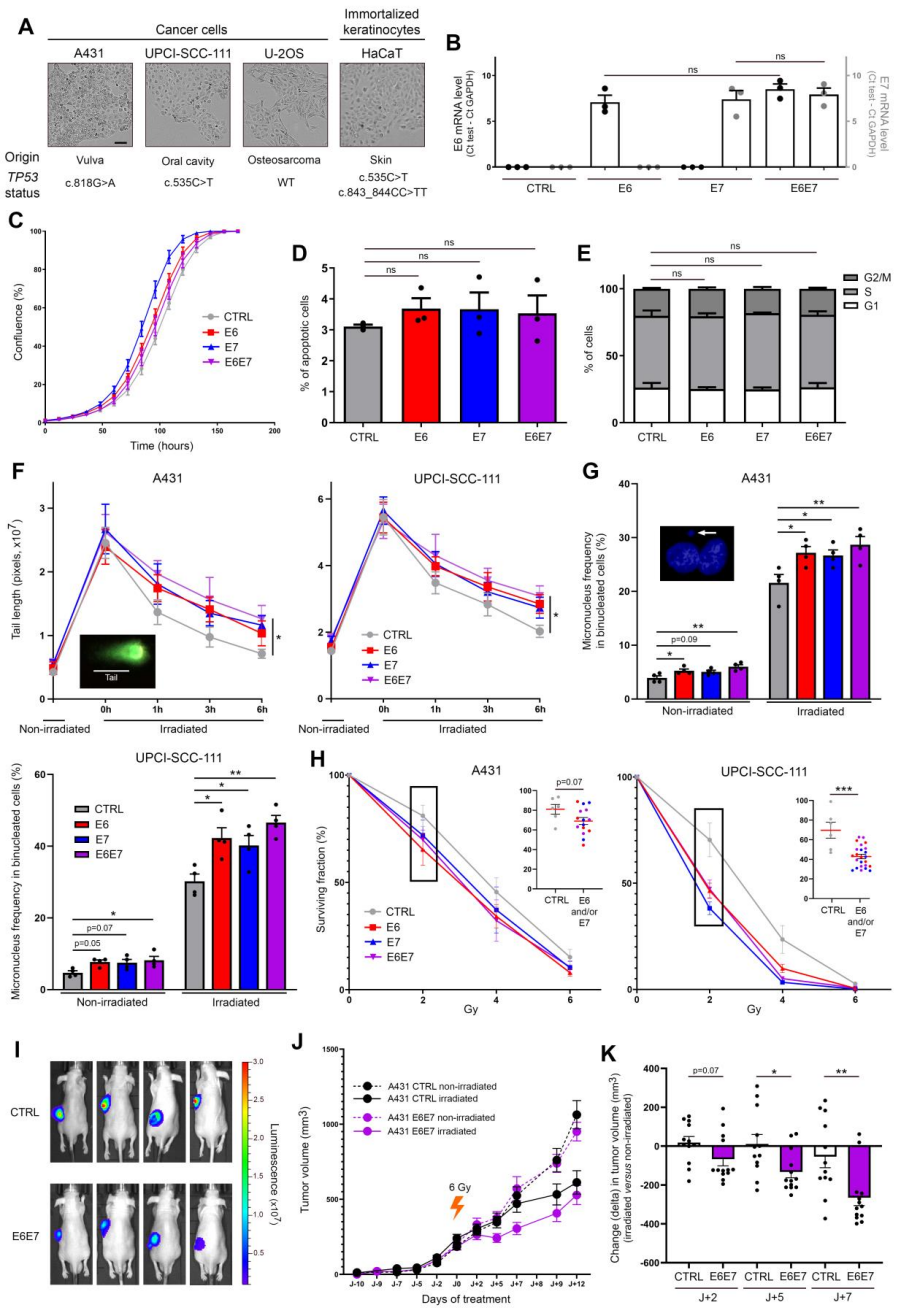
** E6 and E7 viral oncoproteins increase cellular sensitivity to gamma irradiation. (A)** Optical phase-contrast microscopy pictures of the cell lines used for the generation of isogenic models expressing HPV16 E6 and/or E7. The origin as well as the *TP53* mutation status (determined by DNA sequencing) of each cell line are also mentioned. **(B)** The mRNA level of HPV16 E6 (black dots, left y-axis) and/or E7 (grey dots, right y-axis) was determined by RT-qPCR in transduced A431 cells. No significant difference was observed between conditions. The proliferation **(C)**, apoptosis **(D)** and cell cycle **(E)** of A431 cells stably transfected or not with HPV16 E6 and/or E7 was assessed using the IncuCyte live cell analyzing system, annexin V-propidium iodide staining assay and propidium iodide incorporation, respectively. **(F)** Evaluation of DNA double-strand break repair by neutral comet assay. HPV16 E6 and/or E7-transduced A431 and UPCI-SCC-111 cell were exposed to 40 Gy irradiation. At different time points following irradiation (up to 6h), cells were collected and processed for neutral comet assay. To estimate the degree of DNA damage (double-strand breaks), the length of at least 30 comet's tails was measured in each condition. **(G)** Micronucleus frequencies in transduced A431 and UPCI-SCC-111 cells at basal condition and following irradiation (4 Gy). For each independent experiment (n = 4), the presence of micronuclei was assessed in 250 binucleated cells. **(H)** Clonogenic growth analyses of transduced A431 and UPCI-SCC-111 cells following treatment with increasing doses of irradiation (0-6 Gy). At day 10, the percentage of area covered by cell colonies was determined by computerized counting (ColonyArea ImageJ plugin). For each cell line, the non-irradiated condition (0 Gy) was used as control and set to 100%. Results represent the means ± SEM of at least three independent experiments. Each individual data point at 2 Gy are also shown. **(I)** Representative bioluminescence images (at day 9 after irradiation) of A431 (CTRL/Luc and E6E7-transduced) tumor-bearing mice. **(J)** A431 Luc or A431 E6E7 cancer cells were subcutaneously injected in Nude mice. Tumor-bearing mice were then treated or not with a unique dose of 6 Gy. The mean tumor volumes ± SEM are represented. **(H)** Change (delta) in tumor volume between irradiated tumors and their relevant non-irradiated controls. The scale bars represent 100 μm. Asterisks indicate statistically significant differences (*p < 0.05, **p < 0.01, ***p < 0.001, **** *p* < 0.0001). ns: not significant. *P* values were determined using an ordinary one-way ANOVA (B), one-way ANOVA followed by a Dunnett's multiple comparison post-test (D, F, G), χ^2^ test (E) and unpaired t-test (H, K).

In order to determine whether viral oncoproteins actively contribute to the elevated sensitivity of HPV-infected cells to radiation therapy, immortalized keratinocytes (HaCaT) and three cancer cell lines (A431, UPCI-SCC-111 and U-2OS) from different origins and displaying different *TP53* mutation statuses were transduced with HPV16 E6 and/or E7 (Figure 2A). As shown in Figure 2B and Figure S1A, the levels of viral mRNA were close in each isogenic cell model, allowing their further comparison. Moreover, the expression of E6 and E7 (alone or in combination) did not significantly change the cell proliferation, apoptosis and proportion of cells in different cell cycle phases (Figure 2C-E and Figure S1B-D). The cell morphology was not modified by viral oncoproteins either (data not shown). Given that DNA damage detection and repair capacity of irradiated tumor cells have been shown for a long time to contribute to their radiosensitivity, the impact of HPV16 E6 and E7 on DNA damage response was first assessed by neutral comet assay (Figure 2F and Figure S2). The average length of the comet tails predictably peaked immediately after irradiation. Importantly, the kinetics of DNA double-strand break repair was significantly impacted by E6 and/or E7 expression. Indeed, whereas the majority (~80%) of DNA damages seemed to be repaired 6h after irradiation in control condition, a significant delay in the rate of DNA repair was invariably observed in E6 and/or E7-expressing cells (whatever the analyzed isogenic model). In order to expand our DNA damage analysis in presence or absence of HPV oncoproteins, a cytokinesis-block micronucleus assay was carried out. As shown both in Figure 2G and Figure S2, E6- and, at a lesser extent, E7-transduced cells already displayed a slight increase of micronucleus formation compared to their corresponding control cells at basal (non-irradiated) condition. This increased frequency of micronuclei in binucleated cells was even more noticeable following irradiation. In parallel to these two tests directly measuring DNA damages, clonogenic growth analyses were also performed (Figure 2H and Figure S2). Although the statistical significance was not reached with the A431 isogenic models (*p* = 0.07), the expression of E6 and/or E7 viral oncoproteins was consistently associated with an increased cellular sensitivity to irradiation. Using this gold standard method for measuring long-term effects of radiotherapy, it is interesting to notice that a synergistic effect of E6 and E7 was only observed with wild-type *TP53* cells (U-2OS) (Figure S2). Finally, heterotopic tumor models were performed to further assess the radiosensitivity of HPV E6E7-transduced cells. To do so, cancer cells were subcutaneously injected in Nude mice. A431 tumor-bearing animals were then treated or not with a unique dose of 6 Gy when solid tumors (100-200 mm^3^) were established. As shown in Figure 2I-K, following irradiation, the tumor growth delay was significantly more important in E6E7-transduced cancers compared to their control homologs, confirming our *in vitro* data. Of note, *in vivo* experiments using malignant UPCI-SCC-111 and U-2OS cells were also tried. Different cell numbers and conditions (e.g., with or without matrigel) were tested but their weak tumorigenicity (low capacity to develop palpable tumors *in vivo*) did not enable their use in mouse models.

### Both high-throughput screening and co-IP experiments unequivocally reveal the direct interaction between E6/E7 viral oncoproteins and some key proteins involved in DNA damage and repair mechanisms

**Figure 3:**
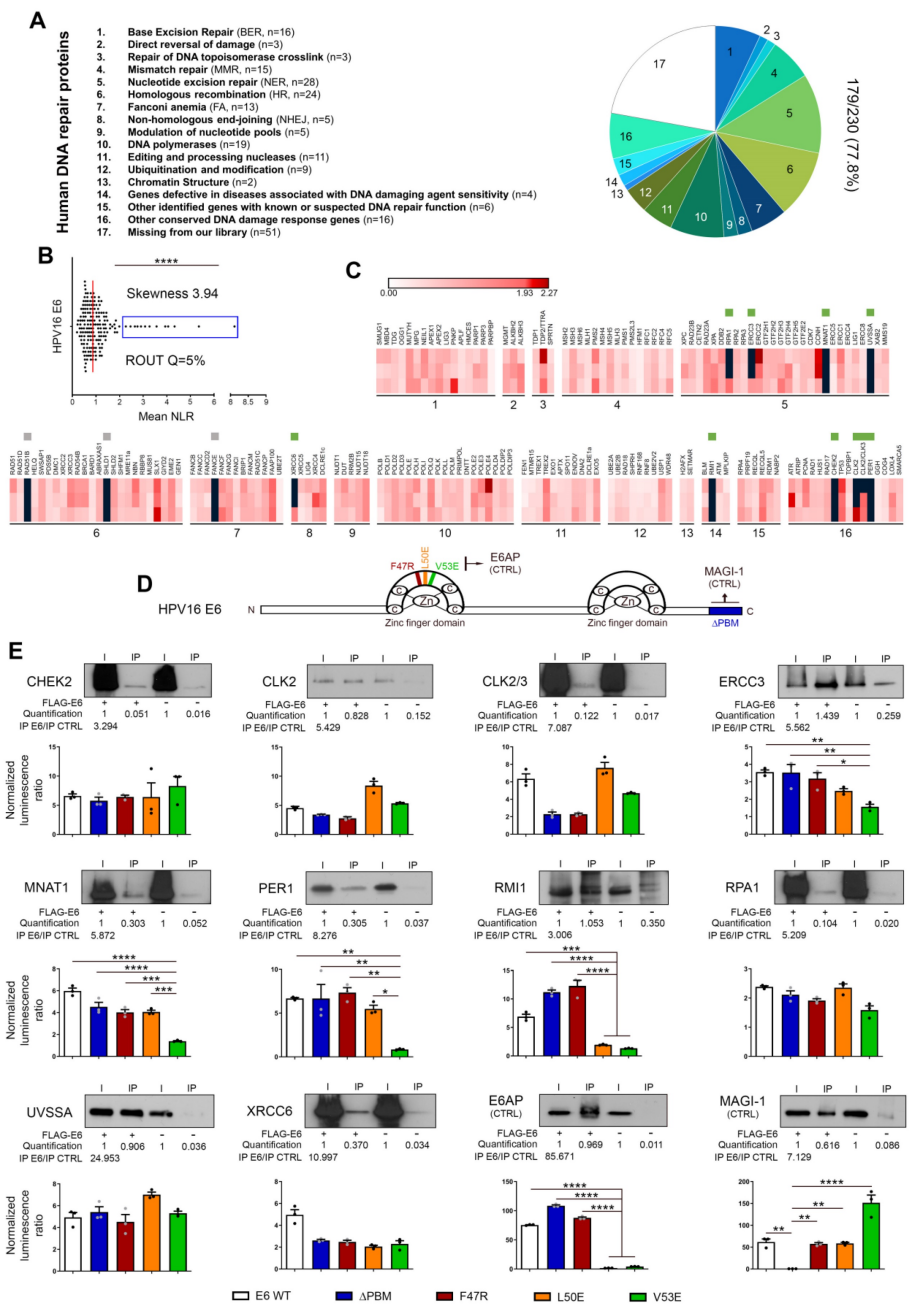
** Bindings of HPV16 E6 with DNA damage/repair proteins: from large-scale screening to validation/characterization. (A)** Pie chart representation of the DNA repair library generated from ORFs contained in the human ORFeome collections v7.1 and 8. According to the Human DNA Repair Gene Database, the 179-cDNA encoding for unique proteins were divided into 16 sub-families. In total, about 80% (179/230, 77.8%) of the entire DNA damage/repair system is represented in the present library. **(B)** Scatter plot of the mean NLR values obtained by GPCA when the library was screened for direct interactions with E6 from HPV16. Confirmed by the skewness score of 3.94, the collected data were not normally distributed and outlier luminescence values (blue rectangle), representing potential interacting pairs, were detected using the ROUT method (with Q=5%). **(C)** Heatmap representing the NLR scores of each protein from the library tested for potential interaction with HPV16 E6. The black blocks represent values above the threshold set for outliers. Green and grey squares represent proteins identified as outliers and confirmed or not (false-positive) by co-IP, respectively. Three biological replicates were performed with each pSPICA-N1 plasmid of the library. The names of each family of DNA repair proteins are listed in the part A of the Figure. **(D)** Schematic representation of the 158 amino acids constituting HPV 16 E6 oncoprotein. In color, the parts that were either truncated (ΔPBM, blue) or mutated (F47R, red; L50E, orange and V53E, green) for the experiments presented in the next panel. **(E)** The protein targets for HPV16 E6 identified by GPCA and validated by co-IP are represented. To further characterize the uncovered interactions, GPCA experiments were also performed using truncated (ΔPBM) or mutated (F47R, L50E V53E) forms of HPV16 E6. E6AP and MAGI-1 were used as positive control for binding to the LxxLL motif and the PBM region, respectively. Results represent the means ± SEM of three independent experiments. Asterisks indicate statistically significant differences (*p < 0.05, **p < 0.01, ***p < 0.001, ****p < 0.0001). *P* values were determined using a D'Agostino and Pearson normality test (B) and one-way ANOVA followed by a Bonferroni post-test (E).

**Figure 4:**
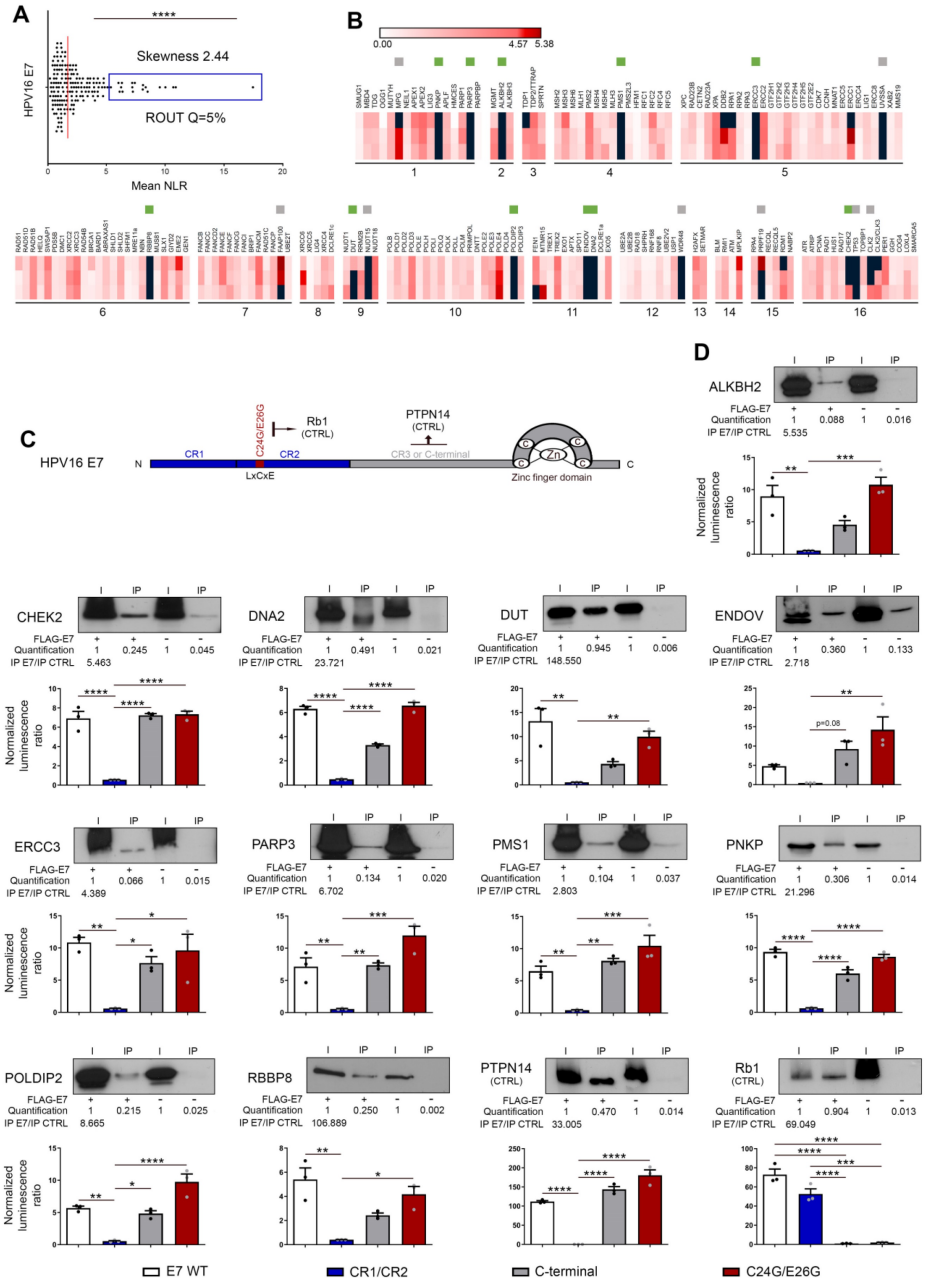
** High-throughput screening and validation/characterization of DNA damage/repair proteins interacting with E7 oncoprotein from HPV16. (A)** Scatter plot of the mean NLR values obtained by GPCA when the library of unique proteins (n = 179) was screened for direct interactions with HPV16 E7. The collected data were not normally distributed (skewness score=2.44) and 19 outlier luminescence values (blue rectangle), representing potential protein-protein interactions, were detected using the ROUT method (with Q=5%). **(B)** Heatmap representing the NLR scores of each protein from the library tested for potential interaction with HPV16 E7. The black blocks represent values above the threshold set for outliers. Green and grey squares represent proteins identified as outliers and confirmed or not (false-positive) by co-IP, respectively. Three biological replicates were performed with each pSPICA-N1 plasmid of the library. The names of each family of DNA repair proteins are listed in the panel A of the Figure 3. **(C)** Representation of the 98 amino acid constituting HPV16 E7 oncoprotein. In color, the parts that were either truncated (CR1/CR2, blue; C-terminal, grey) or mutated (C24G/E26G, red) for the experiments presented in the next panel. **(D)** The protein targets for HPV16 E7 identified by GPCA and validated by co-IP are represented. To further characterize the uncovered interactions, GPCA experiments were also performed using truncated (CR1/CR2 and C-terminal) or mutated (C24G/E26G in the LxCxE region) forms of HPV16 E7. PTPN14 and Rb1 were used as positive control for binding to the c-terminal region and the LxCxE motif, respectively. Results represent the means ± SEM of three independent experiments. Asterisks indicate statistically significant differences (*p < 0.05, **p < 0.01, ***p < 0.001, ****p < 0.0001). *P* values were determined using a D'Agostino and Pearson normality test (A) and one-way ANOVA followed by a Bonferroni post-test (D).

First, a library of cDNA encoding for DNA damage and repair factors was created from ORFs contained in the human ORFeome collections v7.1 and 8.1 (Dana-Farber Cancer Institute, Boston, USA). According to the Human DNA Repair Gene Database (created in 2001 and updated in June 2020, https://www.mdanderson.org/documents/Labs/Wood-Laboratory/human-dna-repair-genes.html), 230 proteins, divided into 16 different families, are directly (or indirectly) implicated in DNA repair mechanisms. As mentioned by the authors (Wood RD and Lowery M), it is important to notice that some proteins act in several pathways but, for the sake of simplicity, each ORF is only listed once (in its main family) in the present study (Figure 3A). The sequences of 18 DNA repair factors were not present in the different human ORFeome collections and, in total, 212 cDNA encoding for unique proteins were cloned in the pSPICA-N1 vector (used subsequently in GPCA experiments). Due to sequence errors identified by Sanger sequencing, 33 cDNA constructs were excluded. Therefore, our library covers about 80% (179/230, 77.8%) of the entire DNA damage/repair response (Figure 3A, Table S2). Twenty-one cDNA encoding for protein isoforms were added in the final version of our library and this latter was used to perform a systematic high-throughput screening for binary interactions between DNA damage/repair proteins and E6/E7 viral oncoproteins. The findings collected with HPV16 E6 and E7 are shown in Figures 3 and 4, respectively. As demonstrated by a skewness score equal to 3.94 (for E6) and 2.44 (for E7), the luminescence values obtained by GPCA were not normally distributed, suggesting potential protein-protein interactions. Using the ROUT method (with a Q set to 5%) 41, 13 and 19 “outlier” luminescence values, representing potential interacting pairs, were highlighted for E6 and E7 oncoproteins, respectively (Figures 3B-C and 4A-B). In order to validate the data collected by the GPCA approach, co-IP experiments were then performed. Both assays were carried out using HEK-293T cells. Thus, as shown in Figure 3E, the interactions between HPV16 E6 and 10 DNA damage/repair proteins (CHEK2, CLK2, CLK2/3, ERCC3, MNAT1, PER1, RMI1, RPA1, UVSSA and XRCC6) were distinctly confirmed. In parallel, the bindings between E7 and ALKBH2, CHEK2, DNA2, DUT, ENDOV, ERCC3, PARP3, PMS1, PNKP, POLDIP2 and RBBP8 were also ascertained (Figure 4D). Intriguingly, ERCC3 and CHEK2 have been shown to interact with both E6 and E7 viral oncoproteins. Regarding the 11 other potential interacting pairs highlighted by GPCA, no clear confirmation by co-IP was obtained, arguing that they very likely represent false positive results of the screening method (Figure S3). When the 21 protein isoforms contained in our library were compared to their full-length homologs, 3 discriminant findings were observed. The isoform specificity of two interactions (PER1 with E6 and DUT with E7) was proved by co-IP (Figure S4). In order to further characterize all these newly uncovered/validated interactions, 4 truncated/mutated forms of HPV16 E6 [the F47R, L50E and V53E constructs containing point mutations within (or in close proximity to) the LxxLL-binding motif as well as an E6 construct truncated for 4 amino acids within the PDZ-binding motif (ΔPBM)] and 3 of HPV16 E7 [the CR1+CR2 region consisting of the 36 first amino acids, the C-terminal domain (37-98 amino acids) and a mutated construct within the LxCxE motif (C24G/E26G)] were also tested. While ERCC3, MNAT1, PER1 and RMI1 seem to interact with the LxxLL-binding motif of E6 (as demonstrated by the low GPCA signal obtained with the L50E and/or V53E construct), the interaction of the other proteins was unaffected by the mutations/deletions within both the LxxLL and PDZ-binding motifs (Figure 3E). Regarding all DNA damage/repair proteins interacting with E7, the GPCA signals were radically reduced when the CR1+CR2 construct was used, supporting their bindings with the C-terminal region of this viral oncoprotein (Figure 4D).

### DNA damage/repair proteins are not degraded following their interaction with E6 or E7 but rather, are recruited to the E1/E2 (viral replication) foci

**Figure 5:**
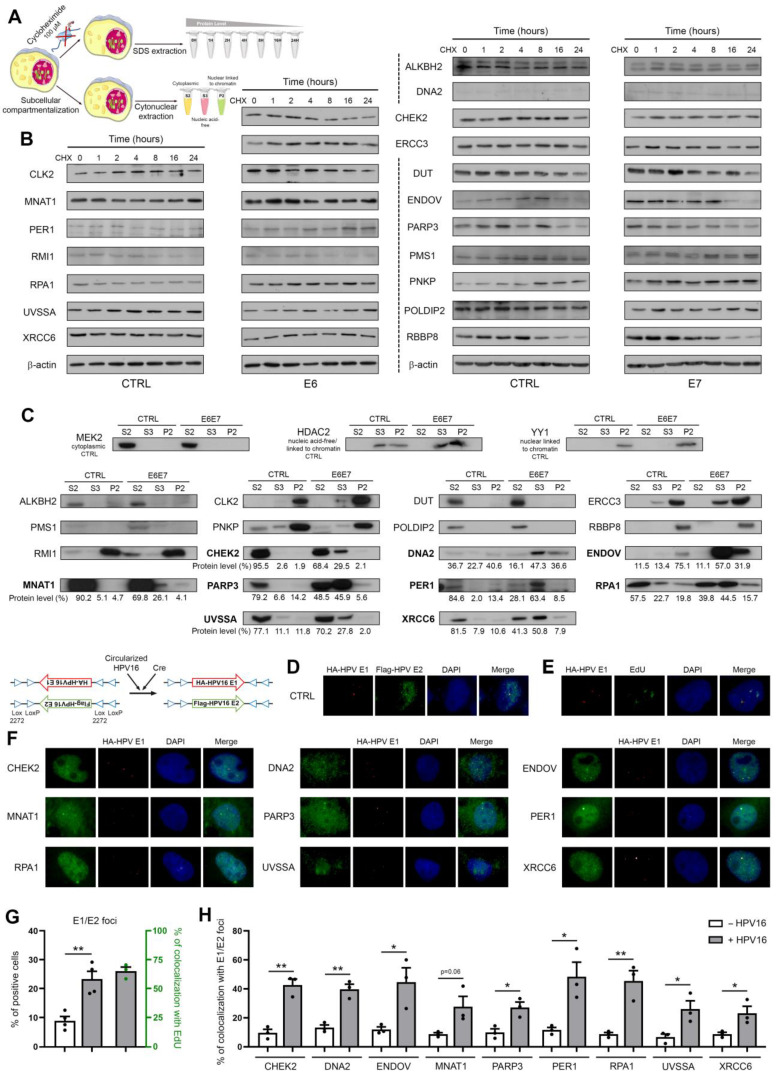
** DNA damage/repair proteins targeted by HPV16 E6 and/or E7 are enriched in the nucleic acid-free protein fraction and partially colocalize with E1/E2 (viral replication) foci. (A)** Schematic illustration of the steps involved in the cycloheximide chase assay and subcellular protein fractionation. **(B)** The isogenic models derived from immortalized keratinocytes (HaCaT) were treated with 100 µM cycloheximide for the indicated times and the stability/half-life of protein targets for HPV16 E6 and/or E7 was determined. A representative immunoblot from at least 2 independent experiments is shown. **(C)** S2 (cytoplasmic), S3 (nucleic acid-free) and P2 (linked to chromatin) protein fractions were collected from HaCaT cells transduced or not with HPV16 viral oncoproteins. The proportions of each newly uncovered protein targets for E6 and/or E7 in S2, S3 and P2 fractions were then assessed by western blot. The protein bands were quantified by densitometric analysis (ImageJ software) and the sum of all three subcellular fractions was set to 100%. MEK2, HDAC2 and YY1 were used as purity control for S2, S3/P2 and P2 fractions, respectively. **(D)** Representative pictures of E1/E2 foci detected in A431 cells expressing both HA-HPV16 E1 and 3xFlag-E2 and transiently transfected with the complete HPV16 genome. To avoid long-term E1 and E2-dependent cellular lethality, the Double-Floxed Inverted Open reading frame technology was used, allowing the expression of both E1 and E2 only following the addition of Cre lentivirus. **(E)** Representative pictures of EdU staining in A431 cells expressing both HPV16 E1 and E2 and transiently transfected with the complete HPV16 genome. **(G)** Percentage of cells displaying E1/E2 foci in the absence or presence of HPV16 DNA (black dots, left y-axis) and percentage of colocalization with EdU (green dots, right y-axis). The significant increased proportion of positive cells in case of circularized HPV16 transfection as well as the frequent colocalization between E1/E2 foci and EdU staining confirm the active viral replication taking place in these cells. Results represent the means ± SEM of three/four independent experiments. For each replicate, at least 250 cells were analyzed. The colocalization between E1 foci and intense nuclear foci (“hot spots”) for CHEK2, DNA2, ENDOV, MNAT1, PARP3, PER1, RPA1, UVSSA and XRCC6 was then analyzed by immunofluorescence **(F)** and quantified by computerized counting (JACoP ImageJ plugin) **(H)**. Results represent the means ± SEM of three independent experiments. For each replicate, colocalization analysis was performed with at least 100 HPV16 E1 foci. Asterisks indicate statistically significant differences (*p < 0.05, **p < 0.01). *P* values were determined using an unpaired t-test (G, H).

We next sought to determine whether the interaction with E6 or E7 modified the stability and/or cellular sublocalization of DNA damage/repair proteins. To do so, immortalized keratinocytes (HaCaT) transduced or not with HPV16 E6E7 were treated with a translation inhibitor (cycloheximide) and the level of each individual protein was monitored over a 24h period. In parallel, subcellular protein fractionations were performed (Figure 5A). Whereas no difference in term of protein turnover was detected (Figure 5B), the expression of viral oncoproteins has been shown to drastically alter the proportion of 9 proteins (CHEK2, DNA2, ENDOV, MNAT1, PARP3, PER1, RPA1, UVSSA and XRCC6) in the different subcellular fractions (Figure 5C). In particular, these proteins were strongly enriched in the S3 fraction corresponding to the nuclear proteins unbound to the host chromatin. Despite a greater nuclear presence of these proteins of interest at basal (E6E7-negative) condition, similar findings were obtained using cancer (UPCI-SCC-111) cells (Figure S5). To confirm these results (and to further characterize the hijacking of host DNA repair proteins by E6 and/or E7), immunofluorescence experiments were performed using malignant (A431) cells expressing both HA-HPV16 E1 and 3xFlag-E2 (following the addition of Cre lentivirus) and transfected with the complete HPV16 genome. As shown in Figure 5D, the coexpression of E1 and E2 resulted in the formation of defined nuclear E1/E2 foci. Both E1 and E2 proteins were invariably observed in fine granular nuclear patterns and the fluorescent signals were always more abundant/extended for E2.

Importantly, the percentage of cells displaying E1/E2 foci (1 to 8 per cell) was 2.5-fold higher (23.35% *versus* 8.95, *p* = 0.0035) in the presence of HPV16 compared to the control condition lacking viral genome (Figure 5G), supporting that most of these latter are indeed active viral replication centers. The frequent colocalization (>65%) between EdU staining and E1/E2 foci indisputably validated the active viral replication occurring in these cells (Figures 5E and G). Determined by computerized counting, E1/E2 foci partially colocalized with “hot spots” (enriched immunolabellings) for the 9 aforementioned DNA damage/repair proteins and, strikingly, the percentages of colocalization were significantly higher in the presence of HPV16 DNA (Figures 5F and H). Altogether, these data argue for the E6/E7-dependent recruitment of these host proteins to the viral replication foci.

### E6/E7 viral oncoproteins globally alter host genome integrity, enhance the cellular sensitivity to DNA repair inhibitors and positively influence their synergy with radiotherapy

**Figure 6:**
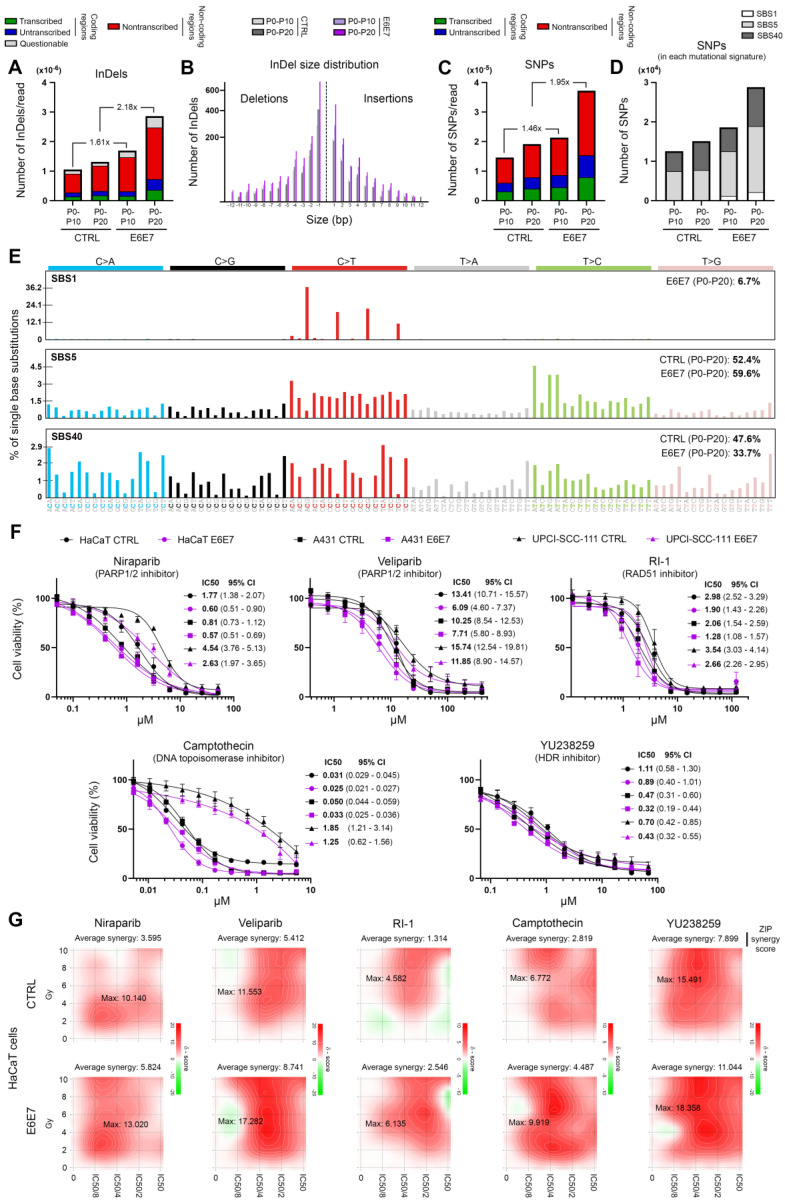
** E6/E7 viral oncoproteins negatively affects host genome integrity and increases cellular sensitivity to various DNA repair inhibitors.** Number of InDels **(A)** and SNPs **(C)** detected in DNA of immortalized keratinocytes (HaCaT) transduced or not with HPV16 E6 and E7. For each time interval (P0-P10 and P0-20), the values were normalized to the total number of sequencing reads. The proportions of InDels/SNPs present in coding or non-coding regions are also represented. Transcribed: the variant is on the transcribed strand. Untranscribed: the variant is on the untranscribed strand. Nontranscribed: the variant is in a non-coding region. **(B)** InDel size distribution (insertions: size >0; deletions: size <0). **(D)** Relative contribution of the different published SBS signatures (COSMIC v3.3) to the overall detected mutational profile. SBS5 (potentially associated to a deficiency of nucleotide excision repair and/or aging) and 40 (unknown etiology) were responsible for >90% of the mutational burden. The COSMIC SBS1 signature (characterized by C>T mutations) exclusively detected in E6E7-transduced cells should be noticed. **(E)** Mutational spectrum of COSMIC signatures of interest (SBS1, 5 and 40). **(F)** Dose-response curves of several HPV E6E7 isogenic cell pairs treated with Niraparib or Veliparib (PARP1/2 inhibitors), RI-1 (RAD51 inhibitor), YU238259 (homology-dependent DNA repair inhibitor) and Camptothecin (DNA topoisomerase I inhibitor) for 72h. Results represent the means ± SEM of four independent experiments. The IC50 and 95% CI of each condition is also indicated. **(G)** 2D synergy maps highlighting synergistic dose regions (in red). The indicated average synergy score for a specific treatment combination was averaged over all the dose combination measurements. The means of three independent experiments were used to assess the synergy between radiotherapy and each DNA repair inhibitor according to the ZIP method.

In order to determine whether E6E7-dependent hijacking of DNA repair pathways jeopardizes host genome integrity, whole genome sequencing experiments were performed. Using HPV16 E6E7 and control isogenic cell pairs generated from immortalized keratinocytes (HaCaT), the number of SNPs and InDels was first estimated at different time intervals. In parallel, the COSMIC mutational signatures were extracted. As shown in Figures 6B and 6D, with the exception of the relatively modest emergence of the Single Base Substitution (SBS) Signature 1 (characterized by C>T mutations), the presence of viral oncoproteins modified neither the relative contribution of each distinct COSMIC signature to the overall mutational profile nor the InDel size distribution. No difference in terms of proportion of InDels/SNPs present in coding or non-coding regions was noticed either (Figure 6A and 6C). The features of extracted COSMIC reference signatures (SBS1, 5 and 40) are detailed in Figure 6E. In contrast, strikingly, E6E7 expression was associated with a substantial increase of both SNPs and InDels in host genome (Figures 6A and 6C), sustaining that the disruption of host DNA repair mechanisms by viral oncoproteins is not harmless for infected cells (and very likely participates to HPV-related carcinogenesis). Given the higher frequency of non-repaired genetic alterations detected following E6E7 expression, we decided to determine whether this feature can be exploited therapeutically. To do so, our HPV16 E6E7 isogenic cell pairs were first treated with several potent drugs targeting the DNA repair machinery [Niraparib and Veliparib (PARP1/2 inhibitors), RI-1 (RAD51 inhibitor), YU238259 (homology-dependent DNA repair inhibitor) and Camptothecin (DNA topoisomerase I inhibitor)]. Interestingly, E6E7-expressing cells were invariably associated with an increased sensitivity to these DNA repair inhibitors compared to their corresponding controls, as demonstrated by the 24 to 295% decrease of IC50 values in case of viral oncoprotein expression (Figure 6F). Despite some intercellular variations, the difference in sensitivity between E6E7-positive and -negative cells was especially noticeable with compounds targeting PARP or RAD51. The synergy between these compounds and radiotherapy was then evaluated in both control and E6E7-transduced keratinocytes (HaCaT cells). As shown in Figure 6G, some DNA repair inhibitors (Niraparib, Veliparib and YU238259) demonstrated clear synergistic effects with radiotherapy (as indicated by maximum ZIP scores >10) while others (especially RI-1) displayed relatively modest additive effects. Irrespective of these differences, strikingly, the calculated (average and maximum) synergy scores were consistently higher in cells expressing E6 and E7 viral oncoproteins compared to their control counterparts.

## Discussion

In the last decades, multiple studies reported that HPV-positive cancers represent a distinct entity associated with a favorable prognosis. In addition, both the specific mutational signature and the elevated immune infiltration of HPV-driven neoplasms (compared to their HPV-unrelated counterparts) were described 42, 43. Altogether, these findings led to the recent recommendation of a clinical dualistic classification based on the HPV status (for oropharyngeal malignancies and proposed for invasive cancers arising from other anatomical sites). In parallel, the hijacking of DNA damage/repair mechanisms by HPV was proposed by several molecular virology research teams. Indeed, HPV proteins would activate and exploit host repair factors to ensure proper viral replication (for a review, see 17, 44). Therefore, could this phenomenon participate to the high radiosensitivity of HPV-positive tumors and represent their Achilles' heel? The significant enrichments of various gene signatures related to DNA repair detected in HPV-positive cancers, the increase of DNA damage/repair biomarker expression detected at the mRNA and protein levels in infected tumor specimens (irrespective of their origin) as well as the results collected by clonogenic growth analysis using both HPV-related and -unrelated cell lines (18 in total) clearly support this hypothesis. These latter data are consistent with those reported by *in vitro* studies published in the past few years and using mainly oropharyngeal cancer cells 9, 45-48.

Playing a major role in viral replication, the implication of HPV E1/E2 in host DNA damage/repair response manipulation has been thoroughly studied 49-51. In the present study, we focused our attention on both E6 and E7 oncoproteins given that, following viral integration into the host genome (occurring in ~70% cervical cancers and ~50% head and neck SCC), E1 and/or E2 genes are frequently disrupted or completely deleted. In parallel, the E2-dependent transcriptional repression of both E6 and E7 is traditionally impeded in HPV-positive (pre)cancer lesions through the methylation of E2-binding sites (E2BSs) in the viral upstream regulatory region (URR), leading to an enhanced/uncontrolled expression of viral E6 and E7 oncoproteins 52, 53. Therefore, the impact of both E1 and E2 early proteins is presumably very limited and the sensitivity of invasive cancers to radiation therapy probably relies mainly on E6 and/or E7. Without affecting the cell proliferation, apoptosis and proportion of cells in the G2/M cell cycle phase, we showed that the sole addition/overexpression of one viral oncoprotein from HPV16 was able to increase intrinsic cancer cell radiosensitivity by 11% to 45% in three different isogenic cellular models. Very likely related to the pivotal function of p53 in both apoptosis and cell cycle arrest, a synergistic effect of E6 and E7 was only detected with tumor cells (U-2OS) displaying a wild-type *TP53* status. In parallel to clonogenic growth analyses, cytokinesis-block micronucleus assays demonstrated that both E6- and E7-transduced cells already displayed an increase of chromosome breakages compared to control cells at basal (non-irradiated) condition. These interesting observations (exacerbated following irradiation) are in agreement with recent studies analyzing the effect of viral oncoproteins from HPV16 as well as of E6 from cutaneous βHPV8 on genome instability 54, 55. Finally, the significant slowing of DNA double-strand break repair kinetics observed in case of viral oncoprotein expression further supported the E6/E7-dependent increase of cellular radiosensitivity.

Given that neither E6 nor E7 possesses an intrinsic enzymatic activity, they act through their interactions with host proteins. Aiming at determining the interactome of HPV E6 and E7 oncoproteins with the different sub-families of DNA repair factors, a cDNA library covering about 80% of the entire DNA damage/repair responses was assembled and the GPCA was used as the high-throughput screening method. This luminescence-based technology represents a major improvement for the identification of binary protein-protein interaction. Indeed, compared to the currently more popular yeast two-hybrid which recovers less than 25% of total interactions 56, GPCA has been shown to be far more sensitive 57. Moreover, by using mammalian cells (HEK-293T), the post-translational modifications of proteins are unaffected. However, GPCA only identifies direct binary interactions, leaving aside all the interactions requiring a tertiary partner (e.g., HPV E6/E6AP/p53). Detected by GPCA and then validated by co-IP, in total, 10 targets for HPV16 E6 and 11 for E7 were identified. It is interesting to notice that not all of these proteins act in the same signaling pathway, supporting that the virus requires various factors to ensure faithful replication of its genome and, therefore, globally hijacks host DNA damage repair. To the best of our knowledge, CHEK2 (which, like ERCC3, interacts with both E6 and E7) is the only host DNA damage/repair protein highlighted in the present study that had previously been reported as a potential HPV target 58. Although the false-negative rate is presumed to be low, of course, we cannot exclude that a few other proteins have been missed by the GPCA screening approach. Interestingly, these 19 different host factors not only interacted with HPV16 E6 and/or E7 but also with the oncoproteins from other high-risk genotypes (HPV18, HPV33 and HPV39), pointing out the importance of these newly discovered targets for viral life cycle (and indirectly for HPV-related carcinogenesis) (Figure S6).

This assumption was further supported by both the absence of degradation of these DNA repair proteins in presence of viral oncoproteins and their colocalization with E1/E2 foci. Of note, these protein recruitments to viral replication foci should no longer appear in invasive tumors displaying a pure integrated infection. However, given that the interactome of HPV E6/E7 with the DNA damage/repair system is not influenced by the viral status (episomal, mixed, full integrated) of infected cells (viral oncoproteins being still expressed), the negative impact for host DNA should occur in all HPV-related cancers. In order to further characterize the revealed interacting pairs, truncated or mutated forms of the relevant viral oncoproteins were tested. All identified targets of E7 exhibited negative GPCA signals with the CR1+CR2 construct (lacking the last 62 amino acids of E7), confirming that most E7-related interactions required the C-terminal region of the oncoprotein 34. Regarding the protein interactions involving E6, 4 (ERCC3, MNAT1, PER1 and RMI1) were affected by L50E and/or V53E mutations directly in (or in the close vicinity of) the LxxLL-binding motif. The interactions of the 6 other DNA damage/repair proteins were not inhibited by any mutations/deletions within the LxxLL or PDZ-binding motifs.

In conclusion, viral E6 and E7 oncoproteins actively contribute to increase the intrinsic radiosensitivity of infected cells. Beside the high blood vessel and T-cell densities detected within tumor microenvironment, this latter parameter undoubtedly also contributes to the better response to radiation therapy of HPV-positive tumors. Even if all identified E6/E7-interacting DNA damage/repair proteins are not involved in signaling pathways directly activated in response to radiotherapy (e.g., base excision repair and non-homologous end-joining pathways), a DNA repair global weakness takes place in HPV-infected cells. Indeed, without drastically modifying the extracted/detected mutational signatures, the expression of viral oncoproteins was clearly associated with a substantial increase of SNPs/InDels in host genome. Therefore, despite an upregulation of multiple factors involved in various DNA repair mechanisms (Figure S7), these genomic data distinctly support that protein hijacking by both E6 and E7 is extremely efficient and the activation of host signaling pathways primarily benefits the viral genome (and not the cellular DNA). Very likely harmless for host genome in normal-appearing cells containing just a few copies of HPV DNA and "tolerated" by the (pre)cancerous cells harboring 100 to 2,000 episomal HPV copies (or in case of viral integration and subsequent uncontrolled E6/E7 expression), the perturbation/hijacking of many host factors by viral oncoproteins would ultimately lead to an increase of cell death following irradiation. This characteristic, not detected in HPV-unrelated cancers, supports the reduced-dose treatment regimens recently proposed in the context of oropharyngeal HPV-positive tumors 59. Furthermore, the targeted inhibition of DNA repair machinery notably using PARP inhibitors (e.g., Niraparib, Veliparib and Olaparib, currently tested with BRCA1/2^mut^ breast, ovarian and prostate cancers 60, 61) could be especially beneficial for treating HPV-positive cancer patients. Indeed, as demonstrated by the decreased IC50 values in case of E6 and E7 expression, viral oncoproteins (and their concomitant global alteration of host DNA repair) enhance the cellular sensitivity to these increasingly tested drugs. Remarkably, the synergy between DNA repair inhibitors and radiotherapy has also been shown to be positively influenced by E6 and E7. Given the over 300,000 deaths still attributable to HPV per year worldwide, such a strategy combining DNA repair inhibitors and radiotherapy certainly merits further study in a controlled clinical trial.

## Supplementary Material

Supplementary figures, tables 1 and 3.

Supplementary table 2.

Supplementary table 4.

## Abbreviations

DAPI: 4′,6-diamidino-2-phenylindole
DMEM: dulbecco's modified eagle's medium
EdU: 5-ethynyl-2'-deoxyuridine
EVS: empirical variant scoring
GPCA: *gaussia princeps* luciferase complementation assay
GSEA: gene set enrichment analysis
HPV: human papillomavirus
InDels: insertions/deletions
MEM: minimum essential medium
ORF: open reading frame
RLU: relative luminescence unit
RPMI: roswell park memorial institute
SBS: single base substitution
SCC: squamous cell carcinoma
SNPs: single nucleotide polymorphisms
URR: upstream regulatory region
