# Supplementary material for: Human papillomavirus E6/E7 oncoproteins promote radiotherapy-mediated tumor suppression by globally hijacking host DNA damage repair
Source: Theranostics. 2023 Jan 31;13(3):1130–49. doi: 10.7150/thno.78091 (PMC9925306; doi:10.7150/thno.78091)
Supplement: Supplementary file 1 — Supplementary figures, tables 1 and 3. [file thnov13p1130s1.pdf]

Supplementary Figures

Human papillomavirus E6/E7 oncoproteins promote radiotherapy-mediated tumor suppression by globally hijacking host DNA damage repair

Diane Bruyere[1], Patrick Roncarati[1], Alizee Lebeau[1], Thomas Lerho[1], Florian Poulain[2], Elodie Hendrick[1], Charlotte Pilard[1], Celia Reynders[1], Marie Ancion[1], Margaux Luyckx[1], Michael Renard[1], Yves Jacob[3], Jean-Claude Twizere[4], Raphael Peiffer[5], Olivier Peulen[5], Philippe Delvenne[1,6], Pascale Hubert[1], Alison McBride[7], Nicolas Gillet[2], Murielle Masson[8], and Michael Herfs[1]

[1]Laboratory of Experimental Pathology, GIGA-Cancer, University of Liege, 4000 Liege, Belgium
[2]Namur Research Institute for Life Sciences (NARILIS), Integrated Veterinary Research Unit (URVI), University of Namur, Namur, Belgium
[3]Unit of Molecular Genetics of RNA Viruses, UMR 3569, CNRS, Pasteur Institute, University of Paris Diderot, 75015 Paris, France
[4]Laboratory of Signaling and Protein Interactions, GIGA-Molecular Biology of Diseases, University of Liege, 4000 Liege, Belgium
[5]Metastasis Research Laboratory, GIGA-Cancer, University of Liege, 4000 Liege, Belgium
[6]Department of Pathology, University Hospital of Liege, 4000 Liege, Belgium
[7]Laboratory of Viral Diseases, National Institute of Allergy and Infectious Diseases, National Institutes of Health, Bethesda, MD, 20892, USA
[8]Biothechnology Superior School, UMR 7242, CNRS, University of Strasbourg, 67412 Illkirch, France

Figure S1

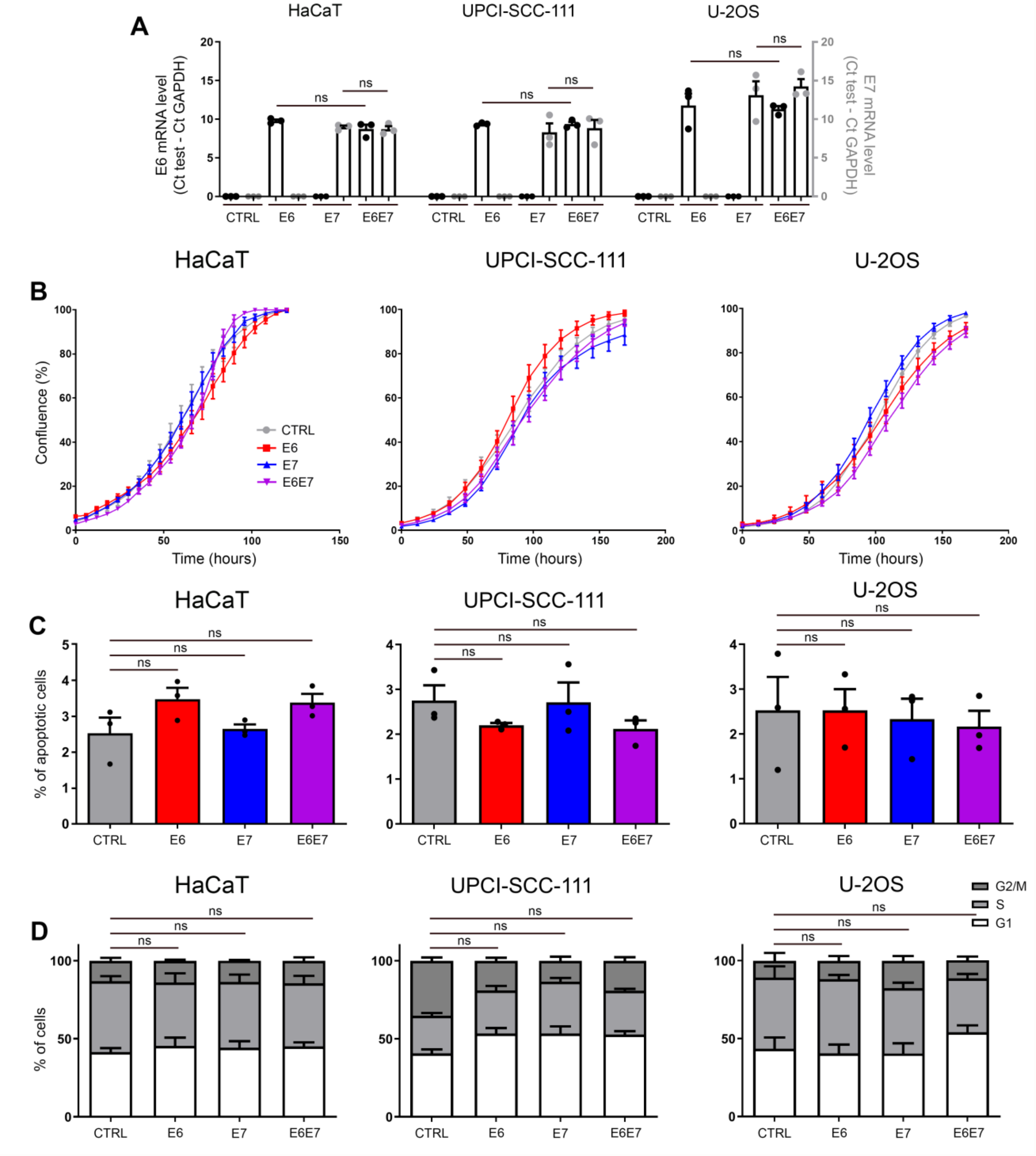

Figure S1. Effect of HPV16 E6 and/or E7 expression on UPCI-SCC-111, U-2OS and HaCaT cell proliferation, apoptosis and cell cycle. (A) The expression of HPV16 E6 (black dots, left y-axis) and/or E7 (grey dots, right y-axis) was determined by RT-qPCR in the different isogenic cell models expressing HPV16 E6 and/or E7. No significant difference was observed between conditions. The proliferation (B), apoptosis (C) and cell cycle (D) of UPCI-SCC-111, U-2OS and HaCaT cells transduced or not with E6 and/or E7 was evaluated using IncuCyte live cell analyzing

system, annexin V-propidium iodide staining assay and propidium iodide incorporation, respectively. Note the absence of impact of viral oncoprotein expression on these three parameters. Results represent the means ± SEM of three independent experiments. The absence of statistical significance (ns: not significant) was determined using an ordinary one-way ANOVA (A), one-way ANOVA followed by a Dunnett's multiple comparison test (C) and χ^2 test (D).

Figure S2

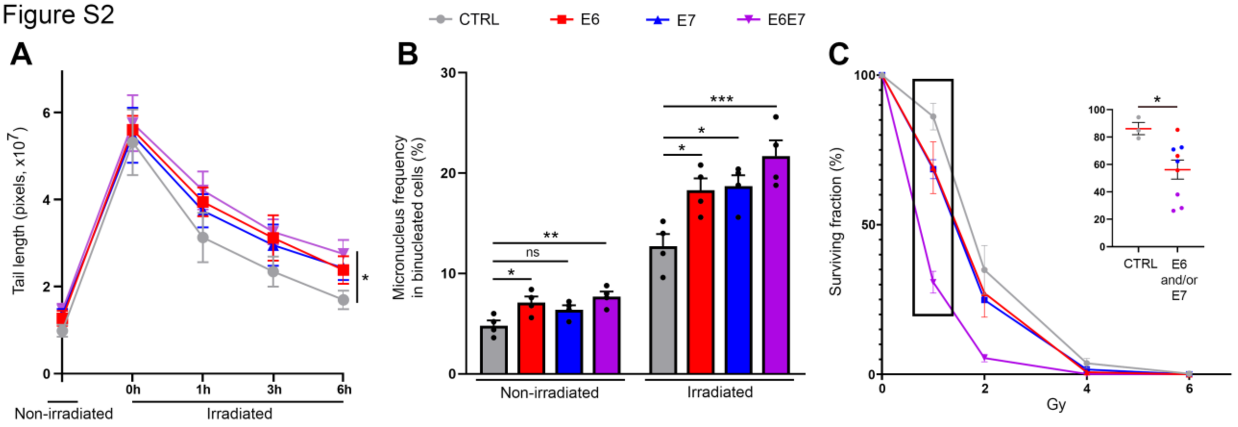

Figure S2. (A) Neutral comet assay, (B) cytokinesis-block micronucleus assay and (C) clonogenic growth analysis using U-2OS cells transduced or not with HPV16 E6 and/or E7. The length of at least 30 comet's tails was measured in each condition. For each independent experiment (n = 4), the presence of micronuclei was assessed in 250 binucleated cells. For the analyses of clonogenic growth, the percentage of area covered by cell colonies was determined by computerized counting (ColonyArea ImageJ plugin) at day 10. The non-irradiated condition (0 Gy) was used as control and set to 100%. Results represent the means ± SEM of three independent experiments. Each individual data point at 1 Gy are also shown.

Figure S3

False positive results of the GPCA screening

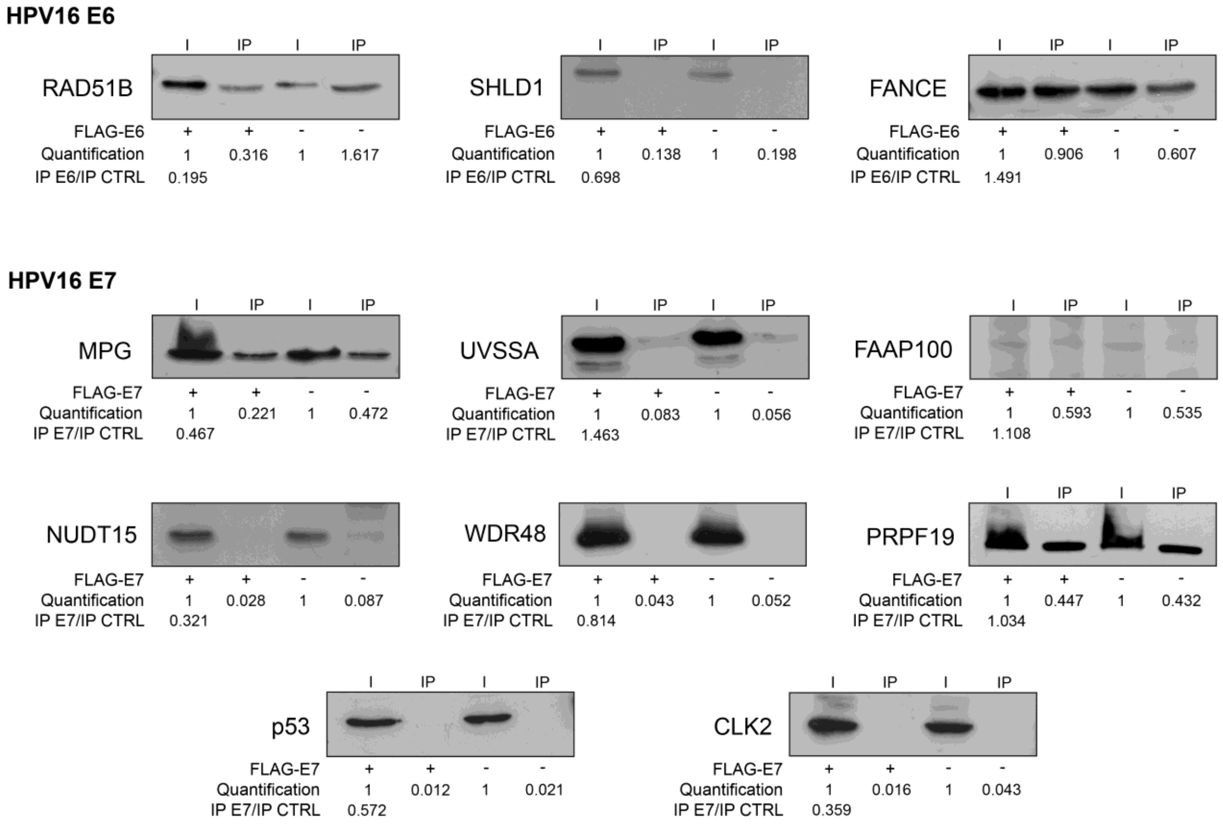

Figure S3. False-positive data of the GPCA screening method. Eleven DNA damage/repair proteins were highlighted by GPCA as potentially interacting with E6 (RAD51B, SHLD1, FANCE) or E7 (MPG, UVSSA, FAAP100, NUDT15, WDR48, PRPF19, p53, CLK2) from HPV16 but the results were not confirmed by co-IP.

Figure S4

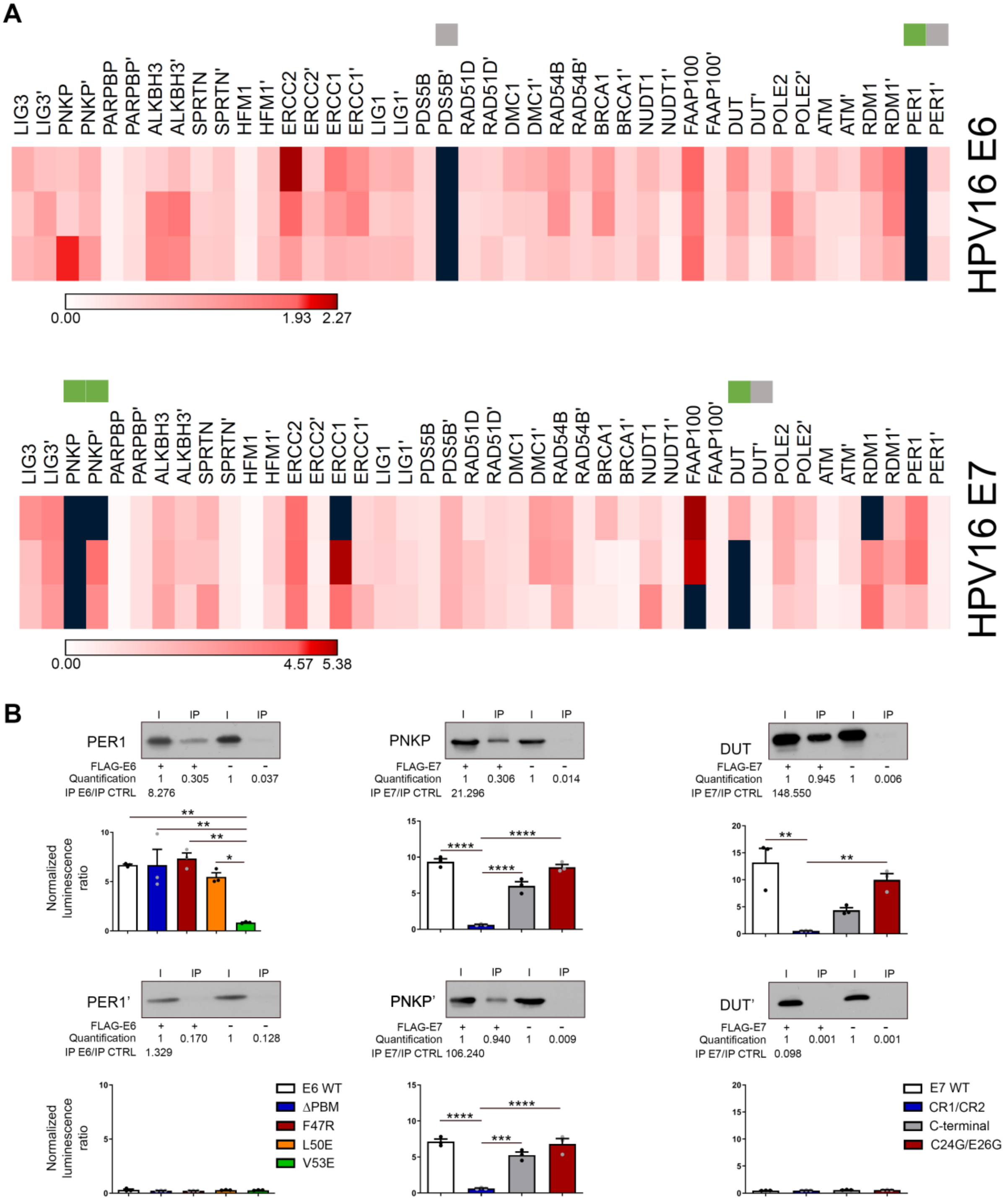

Figure S4. The interactions E6-PER1 and E7-DUT are isoform-specific. (A) Heatmap representing the NLR scores of 21 DNA damage/repair proteins from the library and their respective isoforms tested for potential interaction with HPV16 E6 or E7. Of note, three discriminant results (E6-PER1; E7-PNKP and DUT) were detected by GPCA. The black blocks represent values above the threshold set for outliers. Three biological triplicates were performed

with each pSPICA-N1 plasmid. (B) The isoform specificity of both E6-PER1 and E7-DUT interactions was validated by co-IP. To further characterize these interactions, GPCA experiments were also performed using truncated or mutated forms of HPV16 E6 or E7. Results represent the means ± SEM of three independent experiments. Results collected with full-length proteins were reproduced from Figures 3E and 4D. Asterisks indicate statistically significant differences (*$p < 0.05$, **$p < 0.01$, ***$p < 0.001$, ****$p < 0.0001$). P values were determined using a one-way ANOVA followed by a Bonferroni post-test.

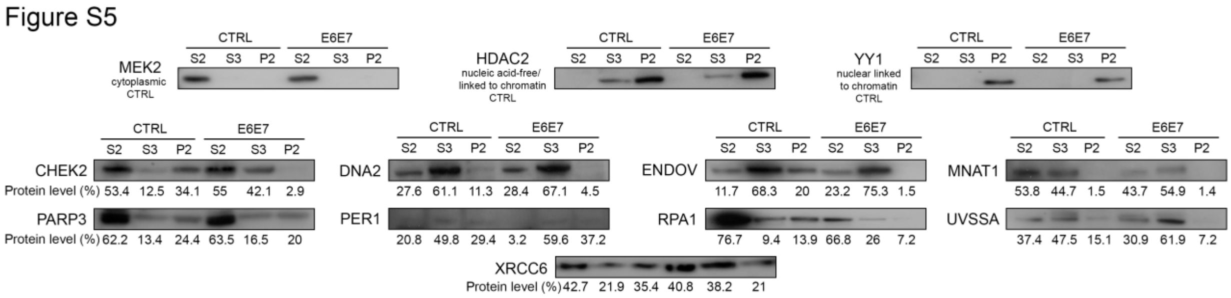

Figure S5. Subcellular protein fractionations (S2: cytoplasmic; S3: nucleic acid-free and P2: linked to chromatin) from oral cancer cells (UPCI-SCC-111) transduced or not with HPV16 viral oncoproteins. The protein bands were quantified by densitometric analysis (ImageJ software) and the sum of all three subcellular fractions was set to 100%. MEK2, HDAC2 and YY1 were used as purity control for S2, S3/P2 and P2 fractions, respectively. With the exception of PAPR3, it is interesting to notice that an enrichment of newly uncovered protein targets for E6 and/or E7 was invariably observed in the S3 fraction (similarly to our data reported with non-cancerous keratinocytes, Figure 5C).

Figure S6

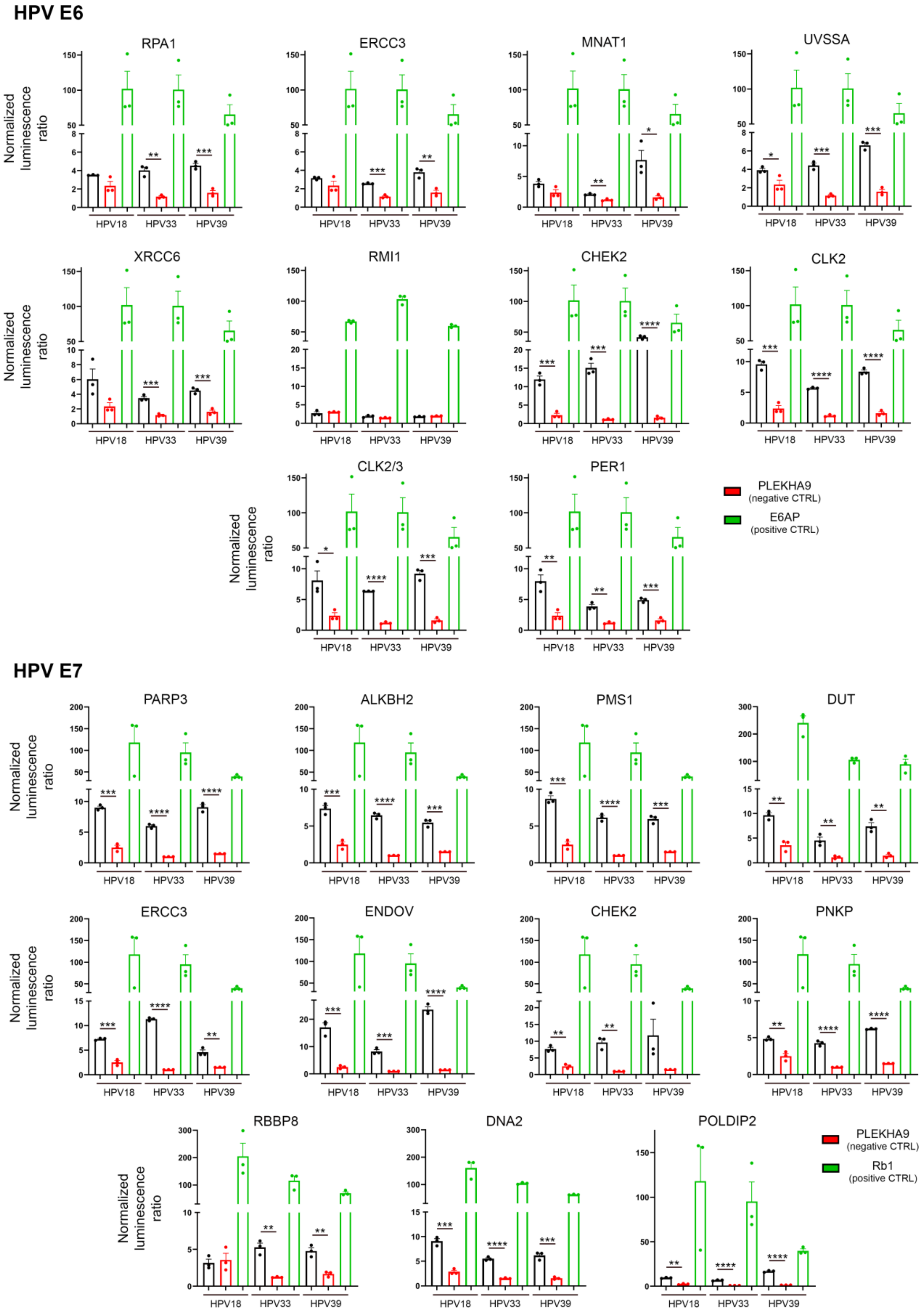
HPV E6
RPA1
ERCC3
MNAT1
UVSSA
XRCC6
RMI1
CHEK2
CLK2
CLK2/3
PER1
Normalized luminescence ratio
HPV18
HPV33
HPV39
PLEKHA9 (negative CTRL)
E6AP (positive CTRL)
HPV E7
PARP3
ALKBH2
PMS1
DUT
ERCC3
ENDOV
CHEK2
PNKP
RBBP8
DNA2
POLDIP2
PLEKHA9 (negative CTRL)
Rb1 (positive CTRL)

Figure S6. Binding of identified targets for HPV16 with E6 or E7 from three other high-risk (carcinogenic) HPV genotypes (HPV18, 33 and 39). The similarity of data collected with HPV16 oncoproteins (see Figures 3 and 4) and their counterparts from other high-risk genotypes should be noticed. E6AP and Rb1 were used as positive control for the GPCA experiments analyzing potential interactions with E6 and E7, respectively. PLEKHA9 was used as a negative control. Results represent the means ± SEM of three independent experiments. Asterisks indicate statistically significant differences (*$p < 0.05$, **$p < 0.01$, ***$p < 0.001$, ****$p < 0.0001$). *P* values were determined using unpaired *t*-tests.

Figure S7

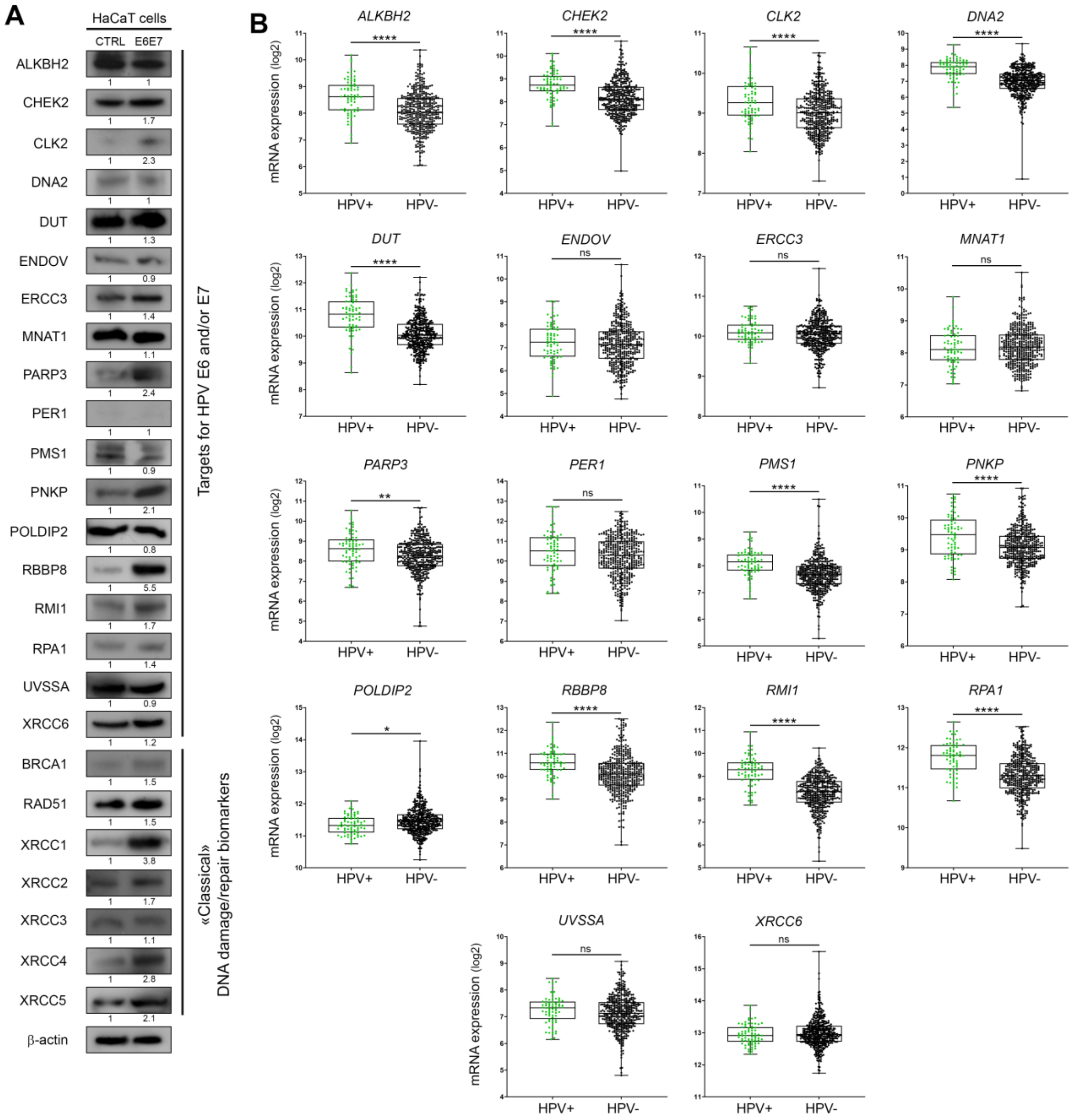

Figure S7. Basal expression level of each novel target for HPV E6 and E7 identified in the present study. (A) Protein extracts from immortalized keratinocytes (HaCaT cells) transduced or not with HPV16 viral oncoproteins (B) and the TCGA public dataset for head and neck SCC were used to assess the protein and mRNA level, respectively.

Table S1. Cell culture conditions used in the present study.

<table>
<tr><th>Name</th><th>Origin</th><th>HPV status</th><th>Culture medium</th></tr>
<tr><td>A431</td><td>Vulva</td><td>HPV-</td><td rowspan="11">Dulbecco’s modified Eagle’s medium containing 10% fetal calf serum, 1% sodium pyruvate, 1% non-essential amino acids (Gibco, Thermo Fischer Scientific, Waltham, MA, USA).</td></tr>
<tr><td>CAL-27</td><td>Base of tongue</td><td>HPV-</td></tr>
<tr><td>SQ-20B</td><td>Larynx</td><td>HPV-</td></tr>
<tr><td>UD-SCC-1</td><td>Oropharynx</td><td>HPV-</td></tr>
<tr><td>FaDu</td><td>Hypopharynx</td><td>HPV-</td></tr>
<tr><td>SiHa</td><td>Cervix</td><td>HPV16</td></tr>
<tr><td>C4-II</td><td>Cervix</td><td>HPV18</td></tr>
<tr><td>HT-3</td><td>Lymph node metastasis from cervical cancer</td><td>HPV30</td></tr>
<tr><td>HeLa</td><td>Cervix (adenocarcinoma)</td><td>HPV18</td></tr>
<tr><td>U-2OS</td><td>Osteosarcoma</td><td>HPV-</td></tr>
<tr><td>HaCaT</td><td>Skin</td><td>HPV-</td></tr>
<tr><td>UPCI-SCC-111</td><td>Oral cavity</td><td>HPV-</td><td rowspan="5">Minimum essential media, supplied with 10% fetal calf serum, 1% sodium pyruvate, 1% non-essential amino acids and 2mM of L-glutamine (Gibco, Thermo Fischer Scientific, Waltham, MA, USA).</td></tr>
<tr><td>UPCI-SCC-40</td><td>Oral cavity</td><td>HPV-</td></tr>
<tr><td>UPCI-SCC-036</td><td>Tonsil</td><td>HPV-</td></tr>
<tr><td>UPCI-SCC-114</td><td>Oral cavity</td><td>HPV-</td></tr>
<tr><td>UPCI-SCC-154</td><td>Base of Tongue</td><td>HPV16</td></tr>
<tr><td>CAL-33</td><td>Base of tongue</td><td>HPV-</td><td rowspan="2">Roswell Park Memorial Institute 1640, supplemented with 10% fetal calf serum, 1% sodium pyruvate, 1% non-essential amino acids (Gibco, Thermo Fischer Scientific, Waltham, MA, USA).</td></tr>
<tr><td>CaSki</td><td>Colon metastasis from cervical cancer</td><td>HPV16</td></tr>
<tr><td>BHY</td><td>Pharynx</td><td>HPV-</td><td rowspan="2">Dulbecco’s modified Eagle’s medium containing 33% Ham’s Nutrient Mixture F12, 10% fetal calf serum, 1% sodium pyruvate, 1% non-essential amino acids (Gibco, Thermo Fischer Scientific, Waltham, MA, USA).</td></tr>
<tr><td>MDA-1483</td><td>Retromolar trigone</td><td>HPV18</td></tr>
</table>

HEK-293T	Embryonic kidney	HPV-	Dulbecco’s modified Eagle’s medium containing 10% fetal calf serum (Gibco, Thermo Fischer Scientific, Waltham, MA, USA).

Table S3. List of primary antibodies used for Western blotting and immunofluorescence.

Target	Manufacturer	Reference	Dilution WB	Dilution IF
ALKBH2	Thermo Fisher Scientific	PA5-21005	1/1000	/
CHK2 (CHEK2)	Cell signaling	6334T	1/1000	1/200
CLK2	Abcam	Ab188141	1/1000	/
dUTPase (DUT)	Abcam	Ab137097	1/1000	/
ENDOV	Thermo Fisher Scientific	PA5-70136	1/1000	1/1000
XPB (ERCC3)	Abcam	Ab190698	1/1000	/
MNAT1	Thermo Fisher Scientific	PA5-49811	1/1000	1/500
PARP3	Thermo Fisher Scientific	PA5-21478	1/1000	1/500
PER1	Biorbyt	Orb374136	1/1000	1/500
PMS1	Thermo Fisher Scientific	PA5-100752	1/1000	/
PNK1 (PNKP)	Bethyl Laboratories	A300-257A-T	1/1000	/
POLDIP2	Thermo Fisher Scientific	PA5-14613	1/1000	/
CtIP (RBBP8)	Cell signaling	9201S	1/1000	/
RPA1	Cell signaling	2267S	1/1000	1/500
KIAA 1530 (UVSSA)	Thermo Fisher Scientific	PA5-28497	1/1000	1/500
Ku70 (XRCC6)	Bethyl Laboratories	A302-623A-T	1/1000	
Ku70 (XRCC6)	Abcam	Ab92450		1/2000
DNA2	Abcam	Ab96488	1/1000	1/500
BLAP75 (RMI1)	Abcam	Ab172625	1/1000	/
BRCA1	Cell signaling	9010T	1/1000	/

RAD51	Abcam	Ab133534	1/1000	/
XRCC1	Cell signaling	76998T	1/1000	/
XRCC2	Santa Cruz biotechnology	SC-365854	1/1000	/
XRCC3	Cell signaling	20026S	1/1000	/
XRCC4	Cell signaling	23908S	1/1000	/
XRCC5	Cell signaling	2180T	1/1000	/
FLAG-Tag	Cell signaling	8146S	/	1/1000
FLAG-Tag	Cell signaling	14793S	/	1/1000
HA-Tag	Cell signaling	2367S	/	1/1000
HA-Tag	Cell signaling	3724S	/	1/1000
